# Predicting conformational ensembles and genome-wide transcription factor binding sites from DNA sequences

**DOI:** 10.1038/s41598-017-03199-6

**Published:** 2017-06-22

**Authors:** Munazah Andrabi, Andrew Paul Hutchins, Diego Miranda-Saavedra, Hidetoshi Kono, Ruth Nussinov, Kenji Mizuguchi, Shandar Ahmad

**Affiliations:** 1 National Institutes of Biomedical Innovation Health and Nutrition, 7-6-8, Saito-Asagi, Ibaraki, Osaka 5670085 Japan; 2 Department of Biology, Southern University of Science and Technology of China, Shenzhen, 518055 China; 3 World Premier International (WPI) Immunology Frontier Research Center (IFReC), Osaka University, 3-1 Yamadaoka, Suita, 565-0871 Osaka Japan; 4 Centro de Biología Molecular Severo Ochoa, CSIC/Universidad Autónoma de Madrid, 28049 Madrid, Spain; 5 Department of Computer Science, University of Oxford Wolfson Building, Parks Road, OXFORD, OX1 3QD, United Kingdom; 6 Molecular Modeling and Simulation (MMS) Group, National Institutes for Quantum and Radiological Science and Technology, 8-1-7, Umemidai, Kizugawa, Kyoto 619-0215 Japan; 7 National Cancer Institute, Cancer and Inflammation Program, Leidos Biomedical Research, Inc. Frederick, Maryland, USA; 8 Department of Biochemistry and Human Genetics, Sackler School of Medicine, Tel Aviv University, Tel Aviv, Israel; 9 School of Computational and Integrative Sciences, Jawaharlal Nehru University, New Mehrauli Road, New Delhi, 110067 India; 10 Faculty of Biology,Medicine and Health, Michael Smith Building, The University of Manchester, Dover Street, Manchester, M13 9PT UK

## Abstract

DNA shape is emerging as an important determinant of transcription factor binding beyond just the DNA sequence. The only tool for large scale DNA shape estimates, *DNAshape* was derived from Monte-Carlo simulations and predicts four broad and static DNA shape features, *Propeller twist*, *Helical twist*, *Minor groove width* and *Roll*. The contributions of other shape features e.g. *Shift*, *Slide* and *Opening* cannot be evaluated using *DNAshape*. Here, we report a novel method *DynaSeq*, which predicts molecular dynamics-derived ensembles of a more exhaustive set of DNA shape features. We compared the *DNAshape* and *DynaSeq* predictions for the common features and applied both to predict the genome-wide binding sites of 1312 TFs available from protein interaction quantification (PIQ) data. The results indicate a good agreement between the two methods for the common shape features and point to advantages in using *DynaSeq*. Predictive models employing ensembles from individual conformational parameters revealed that *base-pair opening -* known to be important in *strand separation -* was the best predictor of transcription factor-binding sites (TFBS) followed by features employed by *DNAshape*. Of note, TFBS could be predicted not only from the features at the target motif sites, but also from those as far as 200 nucleotides away from the motif.

## Introduction

The physical basis of protein-DNA interactions has been explained from the perspective of *direct recognition* of nucleic acid bases by complementary TF residues or through an *indirect recognition* of sequence-dependent DNA structure, more appropriately termed as *base* and *shape* readout respectively in the recent literature^1–4^. While the former ignores the differential accessibilities of DNA bases in the double helix, the latter assumes the existence of a unique and exclusive structure of the DNA. Base readout having been the primary focus of investigations, numerous methods to model sequence features of TF binding sites as a consensus motif or position weight matrices (PWMs) have been successfully developed^5–9^. However, the number of studies focusing on DNA shape or conformational dynamics has been limited^2, 3, 10–13^. Part of the problem is the lack of tools to rapidly estimate the DNA conformation or its dynamics directly from the sequence. The DNA shape prediction tool, “*DNAshape*” provided a major innovation in the computational determination of DNA shape and allowed genome-scale study of the contribution of DNA shape to TF binding site recognition^2^. Trained on Monte Carlo simulation data, *DNAshape* takes a nucleic acid sequence as its input and predicts four sequence-dependent shape features. It uses a five-nucleotide window (for the *base-pair* features) and a six-nucleotide window for the *base-step* features and can be thought of as a dictionary to translate an exhaustive set of small DNA fragments into their corresponding shape features. Use of the four features, predicted by *DNAshape* has resulted in highly accurate classifiers to distinguish between TF binding and non-binding DNA sequences e.g. in explaining the data from the DREAM5 competition for predicting TF binding specificities^14^. Despite these successful implementations and substantial evidence that DNA shape encoded in the sequences is critical for TF recognition, the body of work available on the subject is limited compared to sequence-only analyses. For example, most of the TF target search methods such as *Transfac* or *Uniprobe* have not yet implemented a shape based analysis^15, 16^. Even in the studies employing DNA shape, the focus has been limited to a relatively small number of shape features. It may be intuitive that a simpler model, utilizing fewer features is easier to interpret, but we believe it may not necessarily reflect the entire picture of TF-target recognition. Consequently, it is helpful to explore more shape features and hence verify both the completeness and competitiveness of *DNAshape*. For this purpose, predictive models for much larger set of DNA shape features need to be developed.

We have previously developed techniques to thread DNA sequences onto the structure of a known protein-DNA complex and to determine the energy of cognate and designed sets of sequences^17–23^. This approach was based on developing a statistical force field from the observed co-variances in 12 DNA shape features. Trained *force field* features could estimate the intrinsic sequence energies for a given DNA shape and thereby return the specificities of sequence-dependent structures. Using a combination of base readout energies and those predicted from our statistical force fields, we successfully explained the specificities of the observed DNA sequences in the known protein-DNA complexes. Realizing the inadequacies of the data taken from the crystal structures of protein-DNA complexes, we have also employed molecular dynamics (MD) derived shape data to make more accurate estimates of force field parameters^20^. Separately, we have tried to explore and predict the sequence-dependence of DNA solvent accessibility by analyzing their structures available from the Protein Data Bank (PDB)^24^. However, in most of these works, we focused on shape specificity in terms of sequence-structure relationships and the shape *dynamics* itself was not incorporated into the predictive models. Moreover, our evaluation of sequence specificities was focused on high-resolution structures of protein-DNA complexes and not on the genome-wide TF-target associations.

Here, we present a novel approach, called *DynaSeq* to model DNA sequence specificities using MD-derived sequence-dependent conformational ensembles, instead of their static values. We define and predict an *ensemble* each for an exhaustive set of 13 shape features or conformational parameters, of which 12 (*Shift*, *Slide*, *Roll*, *Buckle*, *Helical Twist*, *Propeller Twist*, *Stagger*, *Shear*, *Tilt*, *Rise*, *Stretch and Opening*) completely describe its atomic structure, and the 13^th^ (*Minor Groove Width;* MGW) is used to draw comparisons with *DNAshape* features^25^. *DynaSeq* is a set of support vector regression models (SVRs), trained to predict conformational ensemble occupancies at different nucleotide positions in a given sequence environment. Ensemble definitions are obtained from a data pool of MD simulation snapshots for 136 unique tetrameric DNA sequences and ensemble occupancies are obtained at each base position in each sequence independently. Subsequently trained models are capable of predicting ensemble occupancies for any nucleotide-sequence in much the same way as *DNAshape* but for a much larger feature set. Upon comparison between *DNAshape* features and the corresponding values derived from *DynaSeq*, we observe a good agreement in the predicted values for the common feature sets. Yet the latter allowed us to investigate the predictability of TFBS from other shape features both as ensembles of single individual parameters as well as a single superset of them all.

For a large scale assessment of the power of the different feature sets of *DynaSeq* (and for comparison, *DNAshape*) to predict TFBS, we utilized the available data set of TFBS for a large number (1312) of TFs identified by the Protein Interaction Quantification (PIQ) algorithm under similar conditions for the same cell type (mouse embryonic stem cells; mESCs)^26^. Developing predictors employing ensemble occupancies of one (out of 13) shape feature at a time we assessed the ability of individual conformational parameters to model TFBS. Results indicated that “*base pair opening*” was the most powerful predictor in majority of the cases followed by some of the features currently employed in *DNAshape*. For models employing a large feature set of all ensemble occupancies of all parameters, *D* *ynaSeq* successfully explained the observed genome-wide binding data with high accuracy. Thus, our results suggest that the performance﻿ of ﻿*DynaSeq* is comparable to and arguably better than the similar models built on *DNAshape* features. Both *DNAshape* and *DynaSeq* based models revealed that in most cases, TFBS could be predicted not only from the shape of the target motifs but also from the flanking regions as far as 200 bases away in a 5′ or a 3′ direction.

Taken together, this study provides a novel approach to study DNA structural dynamics at a genomic scale and indicates that information about TF-DNA binding is contained not only in the exact site of TF-binding but also extends to much larger flanking region of DNA. The dictionary of the current implementatio﻿n of DynaSeq can be accessed from http://dynaseq.sciwhylab.org.

## Results

**Figure 1:**
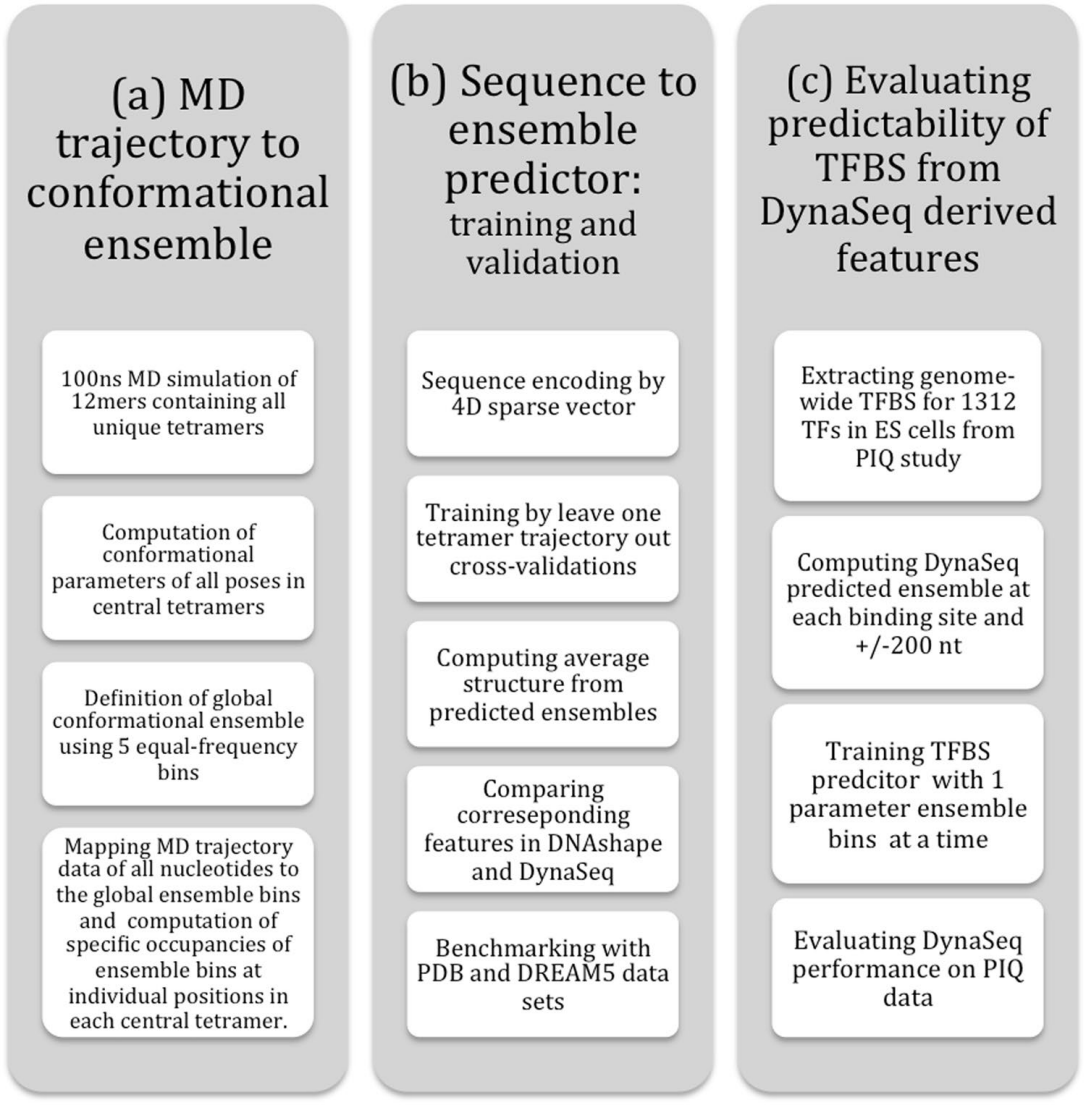
Overall design of the present study. The study consisted of three steps. (**a**) Molecular dynamics (MD) simulations were performed for all the unique tetramers flanked by a fixed tetramer on both terminals and the conformational trajectory of the central four nucleotides was converted into a conformational ensemble by defining equal frequency ensemble bins from the entire data. (**b**) A set of 65 SVR models were trained, one each for the five ensemble bins of the 13 conformational parameters. Models could then use a nucleotide sequence as the input and predict 65 features (representing ensemble bin occupancies) of a nucleotide in the corresponding sequence environment. A number of benchmarks for the effectiveness of *DynaSeq* were performed. These included the models’ performance in recalling PDB deposited structures (using predicted occupancy-weighted averages of ensemble bins) and DREAM5 TF specificities (from the ensemble occupancies for a sequence window). (**c**) Benchmarks on *DynaSeq*’*s* ability to classify TFBS from genomic controls were performed. Predictors were trained by pooling all the 65 features together and also by using just a 5-bin ensemble of a single conformational parameter at a time as the sequence feature.

The overall design of the study is shown in Fig. 1. As shown, the study consists of three components viz. (1) Defining and generating a conformational ensemble for an exhaustive set of tetrameric DNA sequences flanked by a GCGC tetramer on both 3′ and 5′ terminals. (2) Developing and benchmarking *DynaSeq* as a sequence-based tool for predicting shape ensemble occupancies of nucleotides in different sequence environments and finally (3) Applying *DynaSeq* to predict genome-wide binding sites for 1312 TFs and identifying the most predictive shape feature in each of them. Results obtained from these analyses are presented below.

### Global conformational ensemble

*DynaSeq* consists of 65 SVR models trained on Molecular dynamic (MD) simulation data of 136 tetranucleotides, represented by five ensemble bins for each of the 13 helical/step conformational features for each base of the DNA sequence. The observed values of conformational features from the pooled poses of MD trajectories (544 × 10^5^ values) have been used to define five *equal probability* *bins*. The exact ranges or bin boundaries, are shown in Supplementary Figure SF1(a). By definition each bin is expected to have an equal (20%) occupancy, in the global ensembles. However, individual bases and their sequence neighbors, specifically alter these globally deduced values, quantifying the sequence-dependent DNA shape dynamics. General variations in the occupancies across individual ensembles in the MD data (corresponding to various bases under different environments) are plotted in the form of their standard deviations in Supplementary Figure SF1(b). At the first level of specificity, each of the four bases (pooling together all occurrences of the corresponding base irrespective of their flanking bases) has a unique ensemble profile as shown in Supplementary Figure SF1(c). Explicit interpretation of this profile is not possible, but the existence of specific variations, which may play a role in recognizing unknown complementary TF structures, can be noted. For example, the first bin of *shear* is depleted in the case of Adenine and Cytosine and enriched for Guanine and Thymine. On the other hand both of the triple-hydrogen bond forming bases, Guanine and Cytosine, have an enriched first bin for *stretch*, which is opposite to what we observe for Adenine and Thymine. Subtle variations are also observed for other conformational features. In addition to the base-wise variations, sequence neighbors also specifically alter these conformational ensembles, leading to the sequence-dependent DNA conformational dynamics (for reference, specificities caused by a single base neighbor for all 64 possible combinations are shown in Supplementary Figure SF1(d)). Modulation of ensemble bin occupancies by neighboring nucleic acid ﻿bases﻿ forms the basis of *DynaSeq*. Conceptually, this is similar to the sequence-dependent shape features in *DNAshape*. However, the origin of reference data, scope and the content of the two tools are different as outlined above.

### *DynaSeq:* Sequence to ensemble predictor

#### Cross-validation and prediction performance

To develop *DynaSeq* and evaluate its power to predict shape ensembles defined from our MD trajectory data, we created independent training and test data sets in a *leave-one-out* fashion; trained the ensemble occupancies for all base positions in 135 DNA sequences and tested the predictive power for the left-out 136^th^. Results from an exhaustive set of 136 combinations were pooled and evaluated.

**Figure 2:**
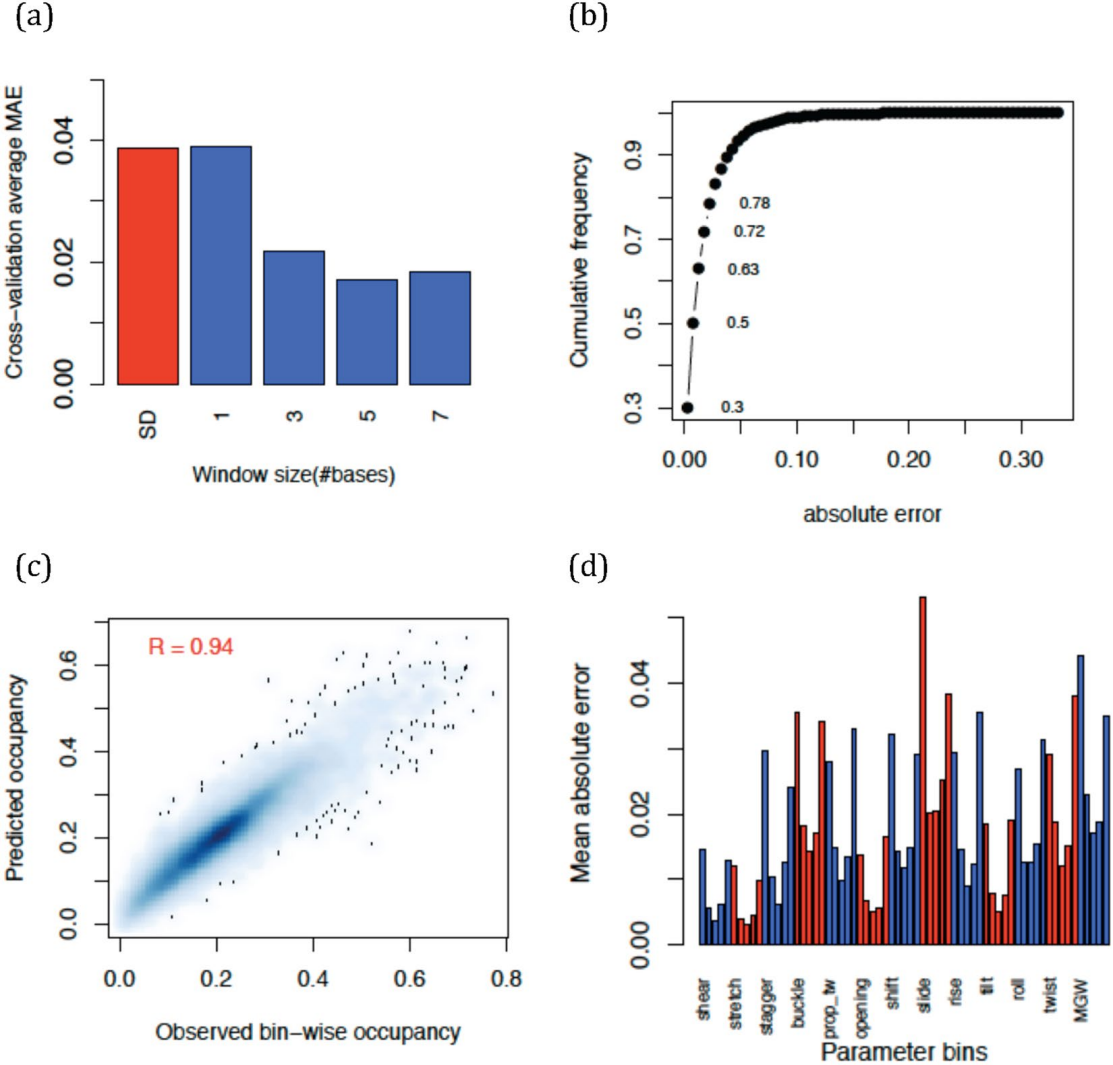
Cross-validation and predictability of DNA conformational ensemble occupancy at each base position. (**a**) Variation of mean absolute error (absolute difference between prediction and observed ensemble occupancy in each bin) with training window sizes. Standard deviation in the overall data is shown in red, whereas other values represent cross-validation performances. (**b**) Overall cumulative frequency of absolute error distribution at window size = 5. Prediction for each base in any position of a tetranucleotide is counted once and errors computed are for the left out sets in leave-one-tetranucleotide cross-validations. (**c**) Scatterplot of predicted versus observed occupancies in all bins and all conformational parameters (**d**) Mean absolute error averages for each bin occupancy.

Each of the 65 SVR models in *DynaSeq*, takes identities (A, C, G or T) of a DNA base and its sequence neighbors within a defined window as inputs and is trained to return the corresponding ensemble bin occupancies as the output. The whole set of 65 occupancy values returned from the models is also referred to as predicted ensemble in the manuscript. Figure 2 summarizes the prediction performance of cross-validated SVR models. We trained and tested various window spans and found that a 5-nucleotide window is optimum for the prediction models on the current data. Based on this optimized model, most of the ensemble bin occupancies are predicted well (~80% with an absolute error of 5 percentage points (Fig. 2b)). A high correlation (R = 0.94) between the predicted and observed values in the entire population range further indicates the stability of the prediction model (Fig. 2c). Furthermore Fig. 2(c) and (d) indicate that the populations, highly skewed from their global 20% average value, are also estimated well, albeit with a slightly higher error rate for the first and last bins than for the values in the middle. However, the worst-case Mean Absolute Error (MAE) is still less than 6% (observed in the first bin of *slide*).

Cross-validation results provided above indicate the robustness of the predictions of sequence-dependent ensembles across 136 independently generated MD trajectories on different unique tetramers. To further evaluate the power of *DynaSeq* models critically, we performed additional benchmarks as described below.

#### Comparison with *DNAshape* on the common descriptors

We have used 3DNA^25^ a widely popular DNA structural analysis tool to describe DNA shape features. 3DNA generates two types of conformational features from the atomic coordinates of a DNA structure: the deformations in complementary bases, also called base-pair features and the deformation with respect to the stacking of base pairs along the helical axis, also called the base-step features. *DNAshape* assigns base-step and base-pair parameters to a pair of bases and an individual base respectively, which is technically appropriate but causes an offset between the two sets of values. To describe a DNA sequence and corresponding shape more uniformly, in *DynaSeq*, we assigned both the base-pair and base-step parameter to a single base position as described in supplementary methods. Thus *DynaSeq* global dictionary for the finally selected 5-base window consists of 4^5^ values, whereas the number is 4^6^ for *DNAshape*.

Further, *DynaSeq* models DNA dynamics instead of its static structures and therefore a comparison with *DNAshape* was made after converting the *DynaSeq*-predicted ensembles to their averaged shape feature values (see Methods).

**Figure 3:**
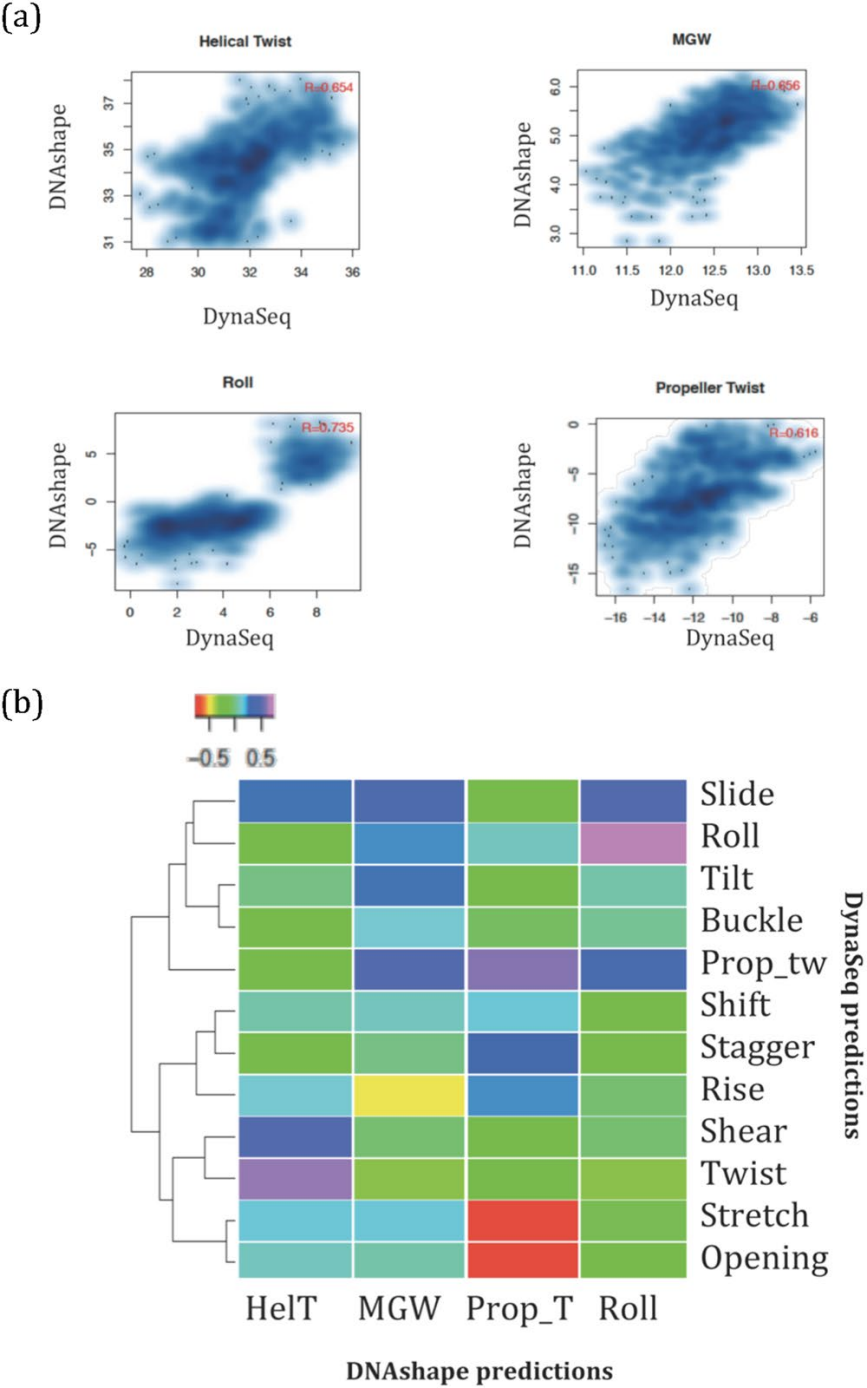
Agreement between *DNAshape* and *DynaSeq* features. The four conformational features provided by *DNAshape* have also been predicted using *DynaSeq* (occupancy-weighted average of ensemble bins). All the four features show strong correlation, supporting the evidence of sequence-dependent specificity in DNA structures. (**a**) Detailed scatterplot of each of the overlapping features. Even though, there is an implicit offset between the MGW values reported in *DNAshape* and *DynaSeq* (due to the use of different definitions and software to compute MGW), the general agreement observed through Pearson’s correlation remains strong. (**b**) Comparison between the 12 *DynaSeq* features and their mutual correlation with the four *DNAshape* features shows that several of the 12 parameters (e.g. shift and tilt) are significantly novel as they show no correlation with any of the *DNAshape* features.

Having addressed the issues described above, we computed *DNAshape* and *DynaSeq* features for all the possible 6-mers (4^6^ = combination; with third base position in *DynaSeq* corresponding to 4^th^ in *DNAshape* for the base-step parameters) and evaluated the agreement between *DynaSeq*-derived equilibrium values and the corresponding *DNAshape* predictions (Fig. 3). We observed a good correlation between the predicted values by the two methods with Pearson’s correlation ranging from 0.61 to 0.73 (Fig. 3(a)). We also observed that several conformational features in *DynaSeq* are not well correlated with *any* of the four *DNAshape* features, thereby suggesting that *DynaSeq* provides more clues about the DNA conformations than *DNAshape* (Fig. 3(b)). However, at the outset it is unclear whether this information, even though different from *DNAshape* is actually useful for modeling transcription factor specificities or otherwise. In the following sections, we present results that highlight what *DynaSeq* could achieve that *DNAshape* could not.

#### Evaluating sequence-specificity of known three-dimensional DNA-structures

DNAshape predicts only four shape features, which are not adequate to model complete DNA structures in atomic details. On the other hand *DynaSeq* is capable of predicting all the parameters required for rebuilding complete three-dimensional structures for DNA and could therefore be useful in docking and other problems of protein-DNA complex design. We evaluated whether the *DynaSeq*-predicted structures of DNA sequences in the protein data banks (PDB) are any closer to the reported structures compared to those predicted for a set of randomly generated sequences of the same length. To avoid, confounding factors from other proteins, we used only the free DNA structures for this purpose. The results indicate that the known DNA structures could be favorably recalled from their predicted ensembles using only the DNA sequence with an RMSD (root-mean square deviation) of 4.2 Å compared to 7.5 Å for the randomly generated sequences (Z-score = −1.48) (summary in Table 1; Detailed results in Supplementary Table ST1). These results provide promise for *DynaSeq*’s ability to help in more accurate design and modeling of protein-DNA complexes^27^.

**Table 1:** Summary of statistics obtained from *DynaSeq*-derived 3D structural models of 115 DNA sequences observed in PDB and 1000 equally sized randomly generated ones.

	Minimum	Q1	Median	Q3	Maximum
PDB RMSD (Å)	1.5	4.1	4.2	4.7	12.8
Random RMSD (Å)	1.9	5.9	7.5	8.9	14.4
Z-score (all PDBs)	−2.79	−1.70	−1.48	−1.26	2.73
P-values (all PDBs)	0.0026	0.0448	0.069	0.103	0.997

Complete distribution for individual sequences is provided in supplementary table ST2.

#### DynaSeq’s ability to predict TF specificities for DREAM5 data sets

Dialogue for Reverse Engineering Assessments and Methods (DREAM) is a series of crowdsourcing challenges to solve biological and medical problems^28^. The fifth of this series of challenges, DREAM5 consisted of a competition to predict TF binding specificities for an exhaustive list of fixed length DNA sequences in a hold-out blind prediction manner^14^. Results of one binding assay were made available to train models and the predictions on a similar assay were tested in a blind experiment. Even though the training and test data sets are somewhat redundant in the two cases, it would be interesting to note how well *DynaSeq* could have performed by doing a retrospective analysis. *DynaSeq* provided highly accurate recall of TF specificities on DREAM5 data sets trained in the manner similar to the conditions of the competition (Supplementary Methods and Supplementary Figure SF2). Prediction was evaluated by AUC (area under the curve) of a receiver operating characteristic curve (i.e. true positive rate plotted against false positive rate), which is used in optimal model selection.

When using the common features in *DNAshape* and *DynaSeq*, AUC results agreed with each other (Figure SF2(a)). When a consensus was taken between results obtained by *DNAshape* and *DynaSeq* models, the AUC shows significant improvement over *DNAshape*-only results with a mean AUC rising from 75.68% to 77.60% (p-value from a paired Student’s t-test = 0.0015) (Figure SF2(b)). Finally, when all the 65 features were used in the prediction model, *DynaSeq* outperformed *DNAshape* by raising the average AUC to 93% (a gain of 18% AUC) (Figure SF2(c)). In particular, *DynaSeq* with the full set of features improved lower AUCs yielded by *DNAshape* (Figure SF2(d)). Some of this improvement could be caused because of high redundancy in DREAM5 experiments and larger number of features in *DynaSeq* compared to *DNAshape*. Even so, in terms of the conditions set out in DREAM5, the proposed *DynaSeq* approach had a clear advantage over *DNAshape* and at the very least provides an alternative route to perform DNA shape analysis at a large scale.

It should be noted that, while this work was being prepared for submission, another study using DREAM5 data for benchmarking *DNAshape* performance in identifying TFBS was reported^29^. However, that work corresponds to a different perspective, demonstrating how *k*-mer information could be combined with shape features and the detailed results about AUC, we employed here are not available. Our focus in the above has been how *DNAshape* and *DynaSeq* predicted features produce similar or different levels of TFBS specificity predictions.

### Predictability of TFBS from *DynaSeq* features in a sliding window

We next set out to evaluate *DynaSeq*’s performance in predicting TFBS using a sliding window on and around the sequence motifs. For this purpose we used the recently reported binding site information for a comprehensive list of TFs identified by a computational algorithm (Protein Interaction Quantitation or PIQ) using DNase-Seq data^30^. A number of attributes of this database make it a good choice to evaluate and compare TF binding site prediction methods. First, the database reports the binding sites of a large number of TFs (1,312). Secondly, binding sites are assigned based on the experimental chromatin accessibility data (DNase-Seq) followed by PWM-based motif assignment, thereby ensuring that the annotated regions are indeed the binding sites of *a given* TF (and not its co-factor, as could be the case in a ChIP-Seq experiment). Thirdly, genome-wide binding and control data come from identical cellular and processing conditions. We evaluated the ability of *DynaSeq*-predicted ensembles to classify TFBS from genomic controls by creating sequence windows at different positions with respect to the motif start position and using their conformational ensembles to create cross-validated classifiers.

We placed a sliding window covering a fixed number of nucleotides at different distances from the motif start positions (motif start site is defined as per the PIQ annotations) and trained an *elastic-net* regularized *logistic regression* model to classify binding sites from non-binding sites (see Methods) using 10-fold cross validation. All the binding sites of a TF and the control regions are collected individually and prediction models are trained for each pair of binding and control regions of the TF. The AUC of ROC for a classifier in this 10-fold cross-validated predictive modeling is used to evaluate the performance of *DynaSeq* features to predict TFBS compared to *DNAshape* features. AUC is determined at all positions for all TFs individually and the entire set of 1312 values is averaged to estimate the performance levels across all TFs.

Prediction models were developed in two ways to draw an unbiased comparison. First, predicted ensemble bin occupancies for only one conformational feature was used and 13 cycles of such predictions were made to assess which of the 13 parameters had the most accurate predictions. Secondly, all the ensembles from all of the 13 parameters were put together to create an all-feature model, primarily to compare it with models trained with the smaller number of static features from *DNAshape*.

#### TFBS predictions from single parameter ensembles

**Figure 4:**
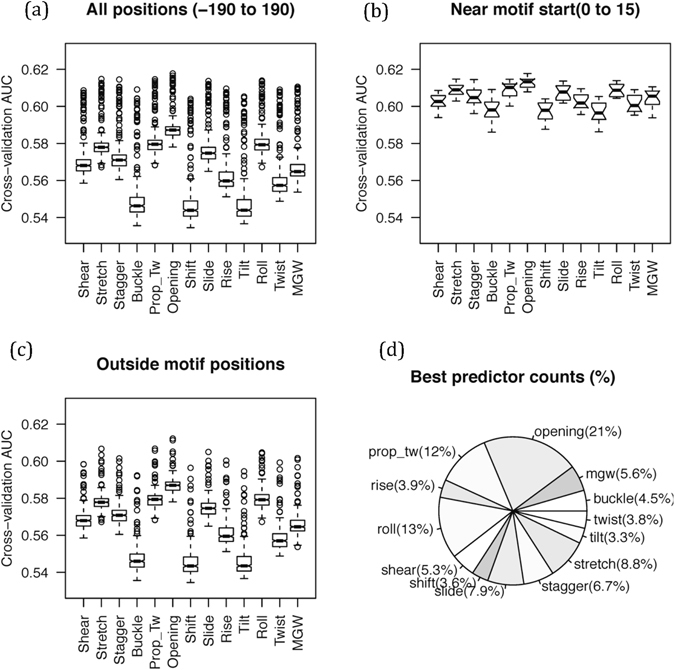
Ability of single parameter conformational ensembles to predict TFBS in PIQ data. (**a**) TFBS’s were classified from genomic control positions using 65 ensemble features at motif site and its +/−200-nt distance using 7-nt window at each position in each of the 1312 TFs and distribution of AUC for all such predictions was represented in a boxplot. (**b**) The data were separated for motif positions (0 to 15 bases from motif start) and (**c**) outside of it. Results in (**a**–**c**) indicated that “*opening*” ensemble is the best predictor of TFBS in both regions (even if the difference between AUC is small), followed by parameters whose static values are also used in *DNAshape* (see (d)) (**d**) Relative number of times a conformational parameter appeared in the top-ranked position in all positions in all TFs was counted.

We examined the performance of each feature by feeding ensembles of only one shape parameter into TFBS prediction models. We observed that even though the ensembles of all the 13 parameters considered here lead to comparable performance levels (Fig. 4a,b), two of the four parameters, *Roll* and *Prop-Tw* used in *DNAshape* are among the top five (Fig. 4d). This validates the choice of the parameters in *DNAshape* and highlights the critical nature of these shape properties. Surprisingly, however, the top ranked feature in our analysis was “*base-pair opening*”, a feature not used by *DNAshape*. It is intuitive to think that *base-pair opening* may represent the DNA base accessibility better than other shape parameters (Supplementary Table ST1). Although detailed experiments will be needed to determine the physical effect of base-pair opening on TFBS, its ability to predict TFBS better than any other single conformational parameter is a surprising outcome of our analysis. It is possible that *base-pair opening* is indeed a critical mechanism of strand separation required for TF binding as argued in earlier works^31^.

From the perspective of accurate prediction of binding sites, it is evident that *DynaSeq* can provide an alternative approach for modeling of genome-wide TFBS.

#### Comprehensive modeling of genome-wide binding sites in 1312 TFs

**Figure 5:**
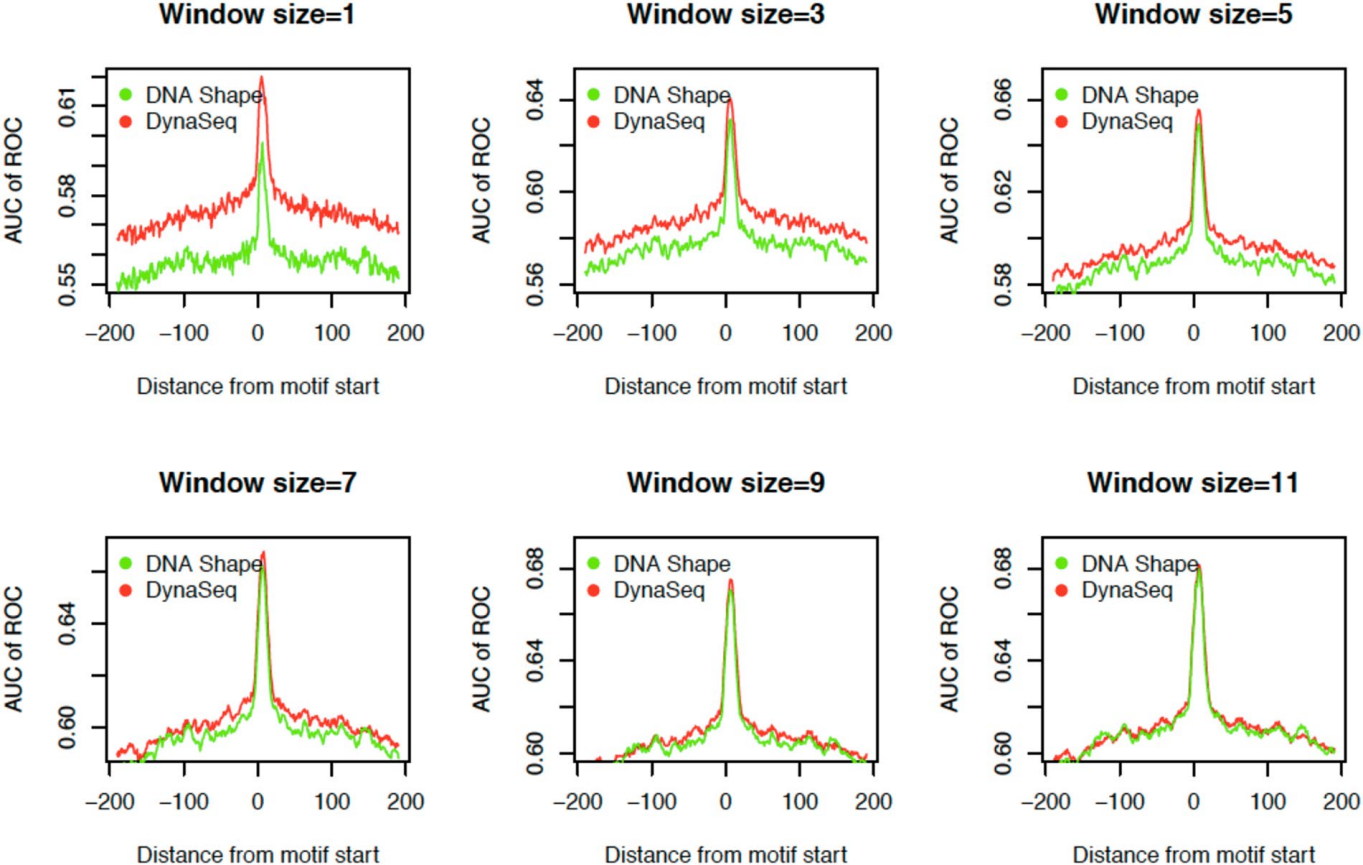
Comparison between *DNAshape* and *DynaSeq* implementations to discriminate TFBS from genome-wide controls. Different sliding windows are placed at the motif start positions and performance levels are scanned +/−200 bases away from motif sites assigned by PIQ. AUC results are computed on 1312 TFs in PIQ data and averaged to produce these plots to have a comprehensive global view. Performance of *DynaSeq* is comparable with *DNAshape* in most cases and seems to be slightly better for smaller window sizes but difference in performances of *DNAshape* and *DynaSeq* diminishes at large window sizes because of strict cross-validation which penalizes models with higher number of features.

To further evaluate how *DynaSeq*-derived shape ensembles can model TFBS dynamics, we created their predictive models from an exhaustive set of features in our models. Figure 5 shows the comparison between prediction models trained using four *DNAshape* features versus those which employ all the 65 bin occupancies used by *DynaSeq* for a range of window sizes. Cross-validation and regularization in the *elastic net* attempts to ensure that the models are not over-fitted for *DynaSeq* (See Supplementary Methods SM2).

We observe that *DynaSeq* could more accurately distinguish binding sites from control as compared to *DNAshape* at the motif position as well as its flanking regions, especially for smaller sliding windows. However, at large window sizes, difference in *DNAshape* and *DynaSeq* performances starts to disappear, presumably because of the use of a strict cross-validation and *elastic net regularization*, both of which penalize models using more features. This is supported by the fact that AUC values for neither *DNAshape*, nor *DynaSeq* based models increase significantly beyond this point. More work would be needed to enable utilization of very large sequence windows for modeling TFBS using *DynaSeq*, although encouragingly *DynaSeq* performs at the same level as *DNAshape* for a window size of 7 nucleotides or smaller. This window size is used for evaluating contributions from an exhaustive set of conformational parameters only few of which are covered in *DNAshape*. Recently, another study also addressed the analysis of the DREAM5 by employing *DNAshape*, they also reported that the models combining *k*-mers with DNA shape are more successful at smaller windows, presumably due to the arguments we present above^29^.

**Figure 6:**
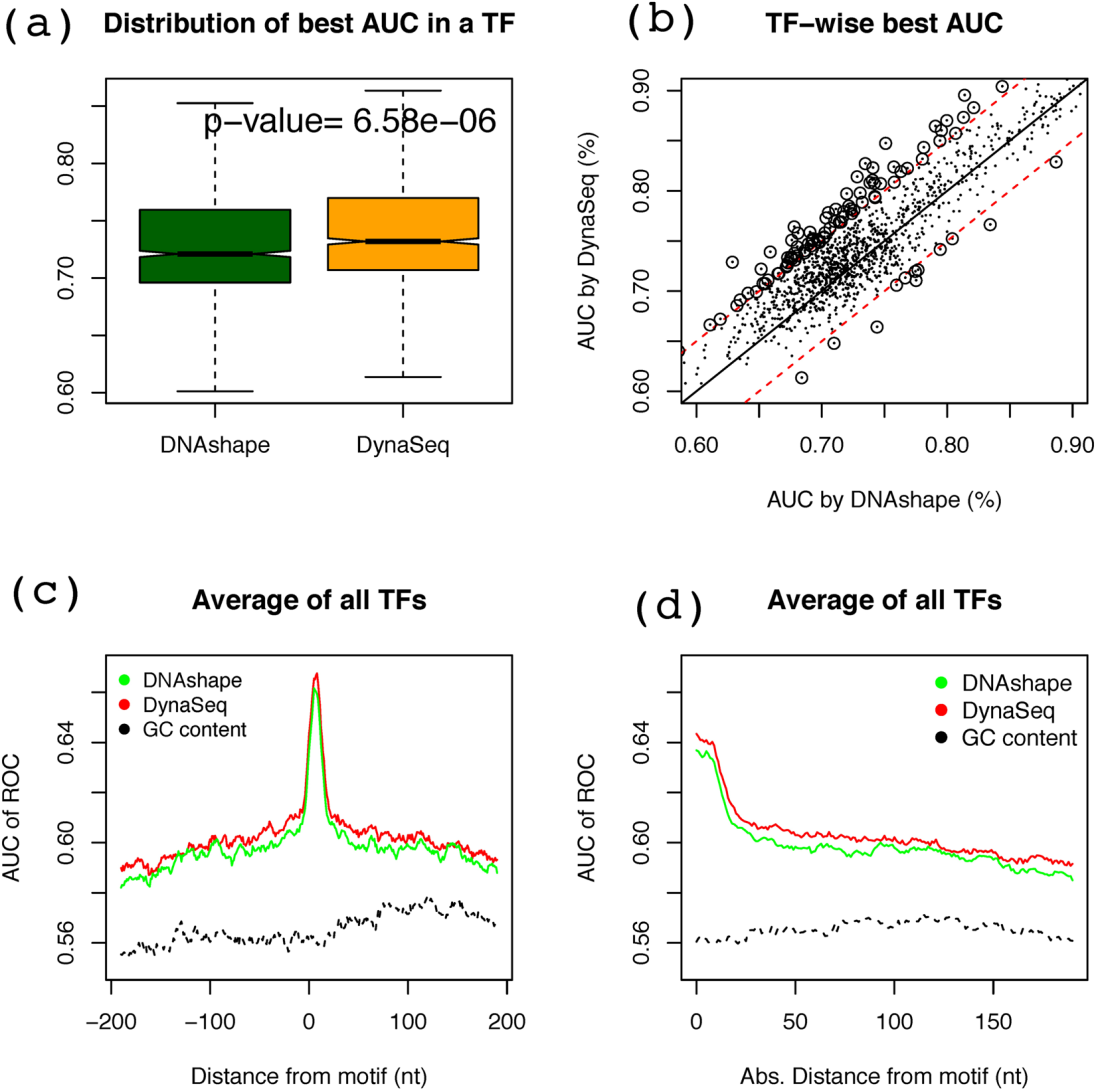
Performance evaluation of DynaSeq for individual TFs and in comparison to DNAshape and GC content based models for a 7-nt window. (**a**) Each TF is represented by a single AUC, which is the highest value from the 401 AUC values computed at each of the +/−200 nt positions from the motif start position of that TF. (**b**) Correlation between the best AUC values by DynaSeq and those by DNAshape. (**c**) A single cumulative performance level is obtained by averaging AUCs of all TFs at each of the 401 positions relative to their motif start site and shows how the perfomance levels vary when DNAshape and DynaSeq features from these positios are used in predictive models. However, such changes are not observed when GC content used. (d) AUC values plotted as a function of distance from motif. The values were calculated using data of Fig. 6(c). The plot gives a directionless estimate of predictability of TFBs from non-motif positions.

With a window size of 7 nucleotides, even though on average the performance levels appear similar, there is still a statistically significant difference in favor of *DynaSeq* in comparison to *DNAshape*. While for reasons discussed above this difference does not hold for larger window sizes, the TF-wise scatterplot of performances (Fig. 6(b)) shows that the general trends are very similar across various TFs. However, 91 TFs show improved performance of more than 5% AUC with *DynaSeq* compared to *DNAshape*, whereas only 12 of them showed the opposite trend. The identities of the two TFs groups are presented in Table 2. A quick look suggests that many of the TFs whose binding sites are better predicted by *DynaSeq* are minor groove binders such as TATA box binding and those known for causing large scale DNA bending in their targets. A comprehensive analysis of their functional implications is being carried out and will be reported separately.

**Table 2:** Transcription factors, which show significantly better (>5% AUC) binding site predictability using (a) DynaSeq than DNAshape features and (b) vice versa of (a).

S. No.	TF ID	TF Name	AUC gain (%)	S. No.	TF ID	TF Name	AUC gain (%)
(a)							
1	MA03841	SNT2	10.0	47	MA01931	Lag1	5.8
2	CN00091	LM9	9.7	48	PB00051	Bbx1	5.7
3	PB00801	Tbp1	9.4	49	PB01791	Sp1002	5.7
4	MA03511	DOT6	9.3	50	MA02591	HIF1AARNT	5.6
5	PL00131	hlh2hlh15	8.6	51	MA02671	ACE2	5.6
6	PH00341	Gbx2	8.6	52	CN00841	LM84	5.6
7	CN00441	LM44	8.2	53	MA03501	TOD6	5.6
8	PH01681	Hnf1b	8.2	54	PB01261	Gata52	5.6
9	PH01641	Six4	8.0	55	PF00101	GCCATNTTG	5.6
10	MA04001	SUT2	7.7	56	PB01321	Hbp12	5.6
11	PB01651	Sox112	7.7	57	PF00561	GGGTGGRR	5.6
12	MA03351	MET4	7.5	58	MA02371	pan	5.5
13	MA01382	REST	7.4	59	MA01421	Pou5f1	5.5
14	MA01551	INSM1	7.3	60	POL0121	TATABox	5.5
15	MA02251	ftz	7.1	61	PB01111	Bhlhb22	5.5
16	MA00831	SRF	7.1	62	MA02321	lbl	5.5
17	CN00271	LM27	7.0	63	MA00411	Foxd3	5.5
18	MA01431	Sox2	7.0	64	PB01631	Six62	5.4
19	PH01071	Msx2	7.0	65	MA04071	THI2	5.4
20	MA00071	Ar	7.0	66	PF01401	RNGTGGGC	5.4
21	PB01981	Zfp1282	6.9	67	PB01751	Sox42	5.4
22	MA03861	TBP	6.9	68	CN02111	LM211	5.4
23	PH01171	Nkx31	6.8	69	CN00521	LM52	5.4
24	PF00231	TAATTA	6.7	70	PB01001	Zfp7401	5.4
25	PL00041	hlh27	6.7	71	MA02861	CST6	5.4
26	PB01571	Rara2	6.7	72	MA01851	Deaf1	5.3
27	PH01701	Tgif2	6.6	73	MA02701	AFT2	5.3
28	PH01161	Nkx29	6.5	74	PF00761	CAGGTA	5.3
29	PL00181	hlh25	6.4	75	MA03141	HAP3	5.3
30	MA01031	ZEB1	6.4	76	PH01731	Uncx	5.2
31	PH01481	Pou3f3	6.3	77	PF00121	CAGGTG	5.2
32	MA00661	PPARG	6.2	78	CN01571	LM157	5.2
33	CN02301	LM230	6.2	79	PF01421	ACAWYAAAG	5.2
34	MA00181	CREB1	6.2	80	MF00031	RELclass	5.2
35	PF01131	AACYN NNNTTCCS	6.2	81	PH00981	Lhx8	5.2
36	MA00601	NFYA	6.1	82	PF00131	CTTTGT	5.2
37	PB01491	Myb2	6.1	83	PH00971	Lhx62	5.2
38	PH01221	Obox2	6.0	84	PB01501	Mybl12	5.2
39	PH01621	Six2	6.0	85	PH01091	Nkx11	5.2
40	CN00821	LM82	6.0	86	PB00081	E2F21	5.2
41	MA01591	RXRRARDR5	6.0	87	CN00591	LM59	5.1
42	PH00701	Hoxc5	5.9	88	MA02581	ESR2	5.1
43	CN01191	LM119	5.9	89	CN02101	LM210	5.1
44	MA04111	UPC2	5.9	90	MA01101	ATHB5	5.0
45	MA00861	sna	5.8	91	MA00792	SP1	5.0
46	PF01631	GGAR NTKYCCA	5.8				
**(b)**							
**S. No**.	**TF ID**	**TF Name**	**AUC gain** (**%**)				
1	MF00071	bHLHzipclass	8.0				
2	MA02771	AZF1	7.0				
3	PH00011	Alx3	7.0				
4	MA01951	Lim3	6.5				
5	PB01861	Tcf32	6.2				
6	PF01191	GGGNRMNNYCAT	5.7				
7	MA01891	E5	5.6				
8	PH00111	Alx12	5.5				
9	CN00361	LM36	5.4				
10	MA01621	Egr1	5.3				
11	MA00131	brZ4	5.3				
12	PB01851	Tcf12	5.2				

#### A comparison with GC-content based predictions

Some shape features such as *propeller-twist* and *opening* have been reported to be highly related with GC-content^30–32^. One wonders if some of the predictive ability in the above models is caused by the GC-content of the binding sites. To address this question, we trained models to predict TFBS using GC content in a 7-nt window for all the considered positions similar to DNAshape and DynaSeq. Figure 6(c,d) shows the results obtained by *DynaSeq*, *DNAshape* and GC-content in the same plot both in terms of the absolute and signed distances from the motif sites. It is clear that at, and even as far as 200 bases from the motif location, the shape-based models carry significantly more information than the GC-content alone. Thus, we conclude that the analysis based on shape and dynamics in this work goes beyond the standard considerations of compositional biases in genomic TF targets. This holds true not only for the composite models with integrated feature sets but also for those based on single conformational parameters as in section 2.3.1 as the latter shows an AUC close to 60%, which is about 5% better than the GC-content based model shown in Fig. 6(c,d).

## Discussion

DNA conformational dynamics is known to play a crucial role in its recognition by proteins. Several approaches to model it from sequence have been developed resulting in increasingly deeper insights^33–39^. The only method for genome-wide prediction of DNA shape available in public domain provides its estimates in terms of static values, which cannot directly capture significant DNA properties such as sequence-dependent polymorphism^38, 39^. In this work, we have shown how the static equilibrium values of *DNAshape* parameters may be augmented with the introduction of a shape ensemble instead of shape parameter values. Further, we include additional parameters that enable modeling the complete atomic structure of DNA for a give sequence.

The exact nature of conformational dynamics in TF recruitment, target search and complex stabilization is not well understood even though the role of binding site proximal and contiguous regions at genomic scales has been recently reported^40^. This study provides support for the results reported in ref. 40 from a different perspective and goes on to look at a more comprehensive description of structure. Conformational dynamics of TFs’ genomic targets has been elusive partly due to the lack of methods to perform large-scale simulations. In this work, we attempt to bridge this gap and show that predicted conformational dynamics provide important biological insights into TF recognition of its genomic targets.

TFs in general show highly redundant sequence-specific DNA binding^41^, yet they can exhibit highly specific cell-type activity^42^. Here we show that DNA regions much larger than the well-known TF binding sequence motifs encode shape and specificity information for TFs, indicating that genomic DNA is not just a ‘passive observer’ of TF binding. Instead, TF-DNA interaction is a mutual event between the DNA sequence and the TF, which acts in unison to bring about specific biological activity, as highlighted earlier^43^. This also reiterates the significance of allostery and cooperativity in protein-DNA recognition as implied from our previous works^44–47^. The allosteric effect in DNA targets in the recognition process is a subject of great interest ^44–46, 48–51^. An analysis at a scale done in this work is not available and our results suggest a role for allosteric control in target recognition of most TF targets.

In this work, we present a novel approach to predict sequence-dependent DNA-conformational ensembles directly from sequences, which does not require detailed simulations of their structures. Models were trained and cross-validated on MD simulations of all unique tetrameric DNA sequences and perform well in various evaluation tests. Model systems representing genome-wide binding preferences of TFs were analyzed. Genome-wide binding preferences of 1312 TFs could be modeled using features derived from predicted conformational ensembles. Together, these results suggest the cooperation of much larger chromatin regions and potential modularity between them for many TF-target associations than realized so far.

*Base-pair opening* is a fundamental molecular process in strand separation which is essential in transcription, DNA replication and recombination^52^. It has been suggested that negative supercoiling of DNA or intermolecular DNA-DNA interactions^33^ induces the strand separation. Thermal fluctuations leading to *base-pair opening*, also called DNA breathing are known to be sequence-dependent^53^, which seems to be well-represented by our conformational ensemble. In this study, we found that the *base-pair opening* feature contributed the most in predicting TFBS, indicating that the energy barrier in the strand separation is lower at TFBS as well as within the 200 upstream and downstream of TFBS. The genomic positions where the strand separation should occur might actually be encoded in the DNA-sequence itself, so that transcription can smoothly start.

In summary, this work presented a novel approach to predict DNA shape in the form of a conformational ensemble and depicted its practical applicability in modeling large scale TFBS data from multiple sources. In particular, the role of DNA shape in protein-DNA interactions is vindicated and a step in understanding them better is made possible.

## Methods

The detailed methods for each one of the three components of this study as outlined in Fig. 1 are explained in the following.

### MD trajectory to conformational ensemble

#### Molecular Dynamic (MD) simulation of 12-mers

*DynaSeq* predictions are based on conformational ensembles obtained by molecular dynamics (MD) simulations. MD simulations were performed for 100 ns on each of the 136 unique (12 mer) DNA sequences with an explicit solvent model. Each of these 12 mers has a unique tetrameric DNA sequences at the center and flanked by GCGC on terminal positions. The detailed simulation conditions in this study were the same as described in our previous work^18^. This method of collecting MD trajectory data to analyze sequence-dependent effects is consistent with our previously reported works^18, 54^ and one by the ABC project^55^. The latter has grown in size and scope over a period of time, but due to practical reasons of data accessibility we retained our method, which has been shown to produce sound scientific insights into sequence-dependent DNA dynamics.

However, as an advance over our previous works, in the current study, we have opted for bsc0 force field to describe nucleic acid atomic interactions^56^ (After this study was completed, another version of this force field *parambsc1* ^57^ was released, which addresses some of the limitations observed in bsc0. Since the approach presented here is based on a high degree of coarse-graining of ensemble populations, we do not expect a dramatic change by using the new version. Notwithstanding, we plan on detailed comparisons that will be reported in the future.) Snapshots were saved every 1 ps (500 steps), yielding 100,000 snapshots for each tetrameric sequence.

#### Computation of conformational features of all poses in central tetramers

From the snapshots obtained above, 13 conformational features at each base position in the central tetramer were computed, leading to 136 × 4 = 544 sets of 13 such values each from a snapshot. A list of 12 of these features (Supplementary Table ST1) and their definitions is provided in Supplementary Materials, while the minor groove width (MGW) forms the 13^th^ feature (not shown in the table). Conformational feature values were computed using a local installation of the *3DNA* program^25^. Both base pair helical and base pair step parameters are adopted from the bp_step.par file generated by the “analyze module” of 3DNA program. As per the convention of this software, base step parameters between the *i-1* ^*th*^ and *i* ^*th*^ positions are assigned to the *i* ^*th*^ position in a nucleotide sequence.

#### Definition of global conformational ensemble using 5 equal-frequency bins

Each of the 13 conformational features from the *snapshots* were pooled together to define five equal frequency bins. Conformational feature values were split into five ranges using four break ﻿points such that each range was occupied by 20% of the *snapshots* or *poses* from all base positions (Figure SF1(a)). For example, all values of twist were pooled together from all positions of all tetramers and sorted in ascending order. Between the extreme observed values and four break points are defined to create five bins, each of which is expected to have 20% poses in a global data. These ranges were termed as global ensemble bins. These occupancies differ from the 20% when data from only specific positions are considered, as seen in (Figure SF1(b–d)). This specificity of different base environments needs to be captured by a training model and forms the basis of most analyses in this work.

#### Mapping MD trajectory data of all nucleotides to the global ensemble bins

As stated above, the individual positions of the 12-mer MD trajectories do not have the same occupancy profile as the global ensemble. To describe the specific divergences from the global values, the ensemble occupancies at each of the 544 base positions in the 136 12-mers were computed. These occupancies were computed in reference to the same global ensemble bins as defined above (employing pooled data) but a 20%-occupancy of in each bin is no longer guaranteed (Figure SF1(b–d)).

### Sequence to ensemble predictor: training and validation of *DynaSeq*

For the purpose of training and cross-validating *DynaSeq*, we used 65 ensemble features on the 544 positions, which form the desired output of a trained model. We described the sequence environment of each of the 544 base positions by the identity of the base at that location and its flanking bases, which formed the input for our training model. Each of the corresponding 65 values can be predicted by one support vector regression (SVR) model. Thus, we created 65 SVR models, each of which takes the sparse-encoded DNA sequence as the input and returns the ensemble occupancy for the central nucleotide position as the output. Once a trained model is ready, a strategy to convert ensemble into averaged structure and comparing predicted values of *DNAshape* and *DynaSeq* for an exhaustive list of 6-mers was developed. These steps are summarized below.

#### Sparse-encoding of DNA sequence

Machine-readable, unique representations of DNA sequences are sparse encoded, as in our previous works on nucleic acids and proteins^24, 58, 59^. In this work each base position (or its *n* ^*th*^ neighbor) is occupied by one of the four bases A, C, G or T. A four-dimensional vector- in which all but one dimension denoting the identity of the base are zero- represents these four possibilities. A DNA sequence segment is a systematic concatenation of these vectors encoding the occurrence of a given base at individual positions.

#### Training and cross-validation of DynaSeq

To ensure that the 544-training instances are not over-fitted, cross-validation was performed by separating trajectories from each of the 136 sequences in the MD data and training the data over 135 cases, and testing how well such models can make predictions for the left-out set of ensemble occupancies. All SVR models were implemented using *e1071* package^60^ in the *R programming environment*, using *RBF kernel* and default *cost* and *gamma* values^61^. Finally a trained model of window size 5 can be thought of as a dictionary of 1024 5-mer sequences mapped to their corresponding 65-dimensional ensemble populations of 13 parameters. This dictionary of the current implementation of *DynaSeq* can be accessed from http://dynaseq.sciwhylab.org as stated in t﻿he abstract.

#### Computing average structure from predicted ensembles

*DNAshape* predicts 4 static values, whereas *DynaSeq* is trained to predict 65 values of the ensemble bin occupancies. To convert ensemble bin occupancies of a given base in a DNA sequence, we used a simple approach of computing the statistical average using the following formula.

$$\langle {C}_{ij}\rangle =\frac{\sum ({M}_{k}{C}_{ijk})}{\sum {C}_{ijk}}$$

where 〈C_ij_〉 is the predicted mean structure for the conformational parameter C at *i* ^th^ base-position in the sequence *j* and *M*_*k*_ and *C*_*ijk*_ are the mid-point values of the *k* ^*th*^ bin and their predicted occupancies respectively.

#### Comparing corresponding features in DNAshape and DynaSeq

To estimate how the predicted values from *DNAshape* compare with *DynaSeq* derived static values defined in this way, we created an exhaustive list of 6-mers (4^6^ in number) and obtained predictions from both tools (see Results). Pearson’s coefficients of correlation between the two predicted values were used to assess the agreement between *DNAshape* and *DynaSeq*.

#### DynaSeq benchmarks on PDB structures and DREAM5 data on TF specificities

In contrast to *DNAshape*, *DynaSeq* predicts all conformational parameters sufficient to rebuild complete three-dimensional structure of DNA. We evaluated if the *DynaSeq* predicted structure of the ‘original’ or ‘native’ sequence from Protein Data Bank (PDB) is closer to its crystal structure, compared to the predictions from random sequences of the same length. Similarly, *DynaSeq* was evaluated if a model trained on DREAM5 training data could correctly recall TF-binding specificities in its test data. Detailed methods to perform both these step and corresponding additional results are provided in the Supplementary Material.

### Evaluating predictability of TFBS from *DynaSeq*-derived features

#### Extracting genome-wide TFBS for 1312 TFs in ES cells from PIQ study

Genome-wide binding sites of 1312 TFs are available from a recent study establishing the directionality of binding in some TFs^26^. Binding site coordinate data was taken from the same study as available from related online resource located at (http://piq.csail.mit.edu/data/v1.3.calls/140906.mES.calls.tar.gz) (data was downloaded on October 1, 2014 and has been reorganized on the authors’ website since then). In the study authors eventually examined the binding sites of only 733 TFs after post processing to discard TFs with insignificant profile strength and merging sets of motifs with similar binding patterns. However, for the present study we have utilized the entire data set of 1312 TFs. Binding sites from both forward and reverse strands are selected from “calls” data and equally sized corresponding controls are sampled at low binding scores assigned by PIQ (0.25 or lower scores; with cutoffs adjusted if the number of reads at this cutoff was too small). Typically the number of binding sites and control in a TF ranges from around 100 to as many as tens of thousands. In the latter case, maximum number of binding sites considered was fixed at 2000, selected by random sampling.

#### Computing DynaSeq -predicted ensembles at each binding site and in the 200 upstream and downstream sequence positions

PIQ data consists of TFs with different motif sizes and there is no natural way to align all of them with respect to one another to develop a cumulative understanding of position dependent predictability of TFBS from DNA shape or dynami﻿cs. Nonetheless, motif start site provides a good reference point as regions following this are enriched in motif residues and away from it are depleted in it. Using motif start site as a reference point, we predicted 65-dimensional *DynaSeq* features at all DNA positions within 200 bases upstream and downstream from the start site. At any given sequence position, where we wish to determine the prediction performance, we utilized these predicted 65 *DynaSeq* features each for all the positions within a window. These features for a window are then concatenated to form the inputs of a new cross-validated prediction model employing *elastic nets*. Similar treatment was given to four-dimensional predictions from *DNAshape* i.e. prediction models for *DNAshape*-based features were also based on the predict shape features for all nucleotide positions within a window.

#### Evaluating DynaSeq performance on PIQ data

Using feature sets and DNA positions as described above, we created new cross-validated prediction models, whose inputs are *shape* or ensemble features of DNA sequence in a window and outputs are class labels indicating if the sequence is derived from a TFBS or a genomic control. For all such binding site prediction models, we used *elastic net regularized logistic regression models*, implemented in the R-package *glmnet* ^62^. Elastic nets provide robust models free from over-fitting with appropriate penalties for large number of features via its adjustable parameter *alpha*, which was selected to be 0.1 in all models being discussed here. In all cases a 10-fold cross-validation was used to estimate strictly independent performance of these models^61^. *Elastic net* implementation in *glmnet* produces multiple prediction models by adjusting a tunable parameter lambda. Out of all such models in each case, we have used the one with highest AUC for all comparisons (see Supplementary Methods SM2).

#### Training TFBS predictor with one parameter ensemble at a time

To assess the extent to which each of the 13 shape features studied in this work contribute to predictions, we repeated the PIQ TFBS prediction steps described above by creating multiple prediction models at each position of each TF. One model each was created for 5-dimensional ensemble of one conformational parameter at one time at each base position in the TFBS of each TF. Average AUC of all TFs for positions enriched in motif (motif start site and a fixed distance on 5′ sequence neighborhood) and remaining regions are considered. In addition, at each base position relative to motif start site, the name of the parameter whose ensemble gave the best prediction performance was retained and finally a frequency of occurrences of each of the 13 feature names corresponding to conformational parameters were compared.

## Electronic supplementary material

Supplementary data
