# Supplementary material for: Predicting conformational ensembles and genome-wide transcription factor binding sites from DNA sequences
Source: Sci Rep. 2017 Jun 22;7:4071. doi: 10.1038/s41598-017-03199-6 (PMC5481346; doi:10.1038/s41598-017-03199-6)
Supplement: Supplementary file 1 — Supplementary data [file 41598_2017_3199_MOESM1_ESM.pdf]

Supplementary material for:

Predicting conformational ensembles and genome-wide transcription factor binding sites from DNA sequences

Munazah Andrabi[1,#], Andrew Paul Hutchins[2], Diego Miranda-Saavedra[3,4], Hidetoshi Kono[5], Ruth Nussinov[6,7], Kenji Mizuguchi[1], and Shandar Ahmad[1,8,*]

1. National Institutes of Biomedical Innovation Health and Nutrition, 7-6-8, Saito-Asagi, Ibaraki, Osaka 5670085, Japan
2. Department of Biology, Southern University of Science and Technology of China, Shenzhen 518055, China
3. World Premier International (WPI) Immunology Frontier Research Center (IFReC), Osaka University, 3-1 Yamadaoka, Suita 565-0871, Osaka, Japan
4. Centro de Biología Molecular Severo Ochoa, CSIC/Universidad Autónoma de Madrid, 28049 Madrid, Spain
5. Molecular Modeling and Simulation (MMS) Group, National Institutes for Quantum and Radiological Science and Technology, 8-1-7, Umemidai, Kizugawa, Kyoto 619-0215 Japan
6. National Cancer Institute, Cancer and Inflammation Program, Leidos Biomedical Research, Inc. Frederick, Maryland, USA
7. Department of biochemistry and Human Genetics, Sackler School of Medicine, Tel Aviv University, Tel Aviv, Israel
8. School of Computational and Integrative Sciences, Jawaharlal Nehru University, New Mehrauli Road, New Delhi-110067, India

* Corresponding author

Supplementary Methods:

Supplementary method SM1:

Definitions of terms used:

Base-pair step at a position *i* in a DNA sequence is defined as the pair of nucleic acid bases at position *i* and neighbor *i+1 towards* its 3' end.

Conformational parameters correspond to 12 unique helical and base-step parameters: *shear, stretch, stagger, buckle, prop-tw, opening, shift, slide, rise, tilt, roll* and *twist*, as provided by the *analyze* option of *3DNA(27)*. These are summarized in Table ST1. Base pair parameters are straightforward to assign to each base position, the base-step parameters, strictly defined for a pair of bases *i* and *i+1* rather than a single base *i* have also been assigned to each base position *i* in this work, primarily to ensure a uniform feature assignment for each base position.

DNA conformational ensemble or just *ensemble* refers to the observed or predicted population density distribution of a single base (and by extension for a DNA sequence) in pre-defined bins (ensemble bins) representing ranges of base-step conformational parameters. One ensemble contains population density data from 12 conformational parameters and is computed from snapshots of the 3D structure in a Molecular Dynamics (MD) trajectory or predicted by a trained model directly from the sequence. There are five bins for each conformational parameter, whose ranges are defined by equal frequency distribution in the global data. Summary of the finally defined ensemble bins and their distribution within each base type as well as divided into different sequence neighbor environments is shown in Figure ST1.

Window size has been used to indicate the sequence-window under consideration in a given model and its actual value depends on the context. Window size values are defined at two levels throughout this work. First the core models in *DynaSeq* are trained and predicted through support vector regression (SVR) models by taking sequence windows of size 0 to 7 and an optimum size of 5-base window was selected. Subsequently all 65-dimensional ensembles at each base position are predicted from this optimum window size (=5). For the purpose of classification of genomic sequences between TF binding sites versus control, sequence windows are varied again. Sequence window in this context means the number of base positions for which *DynaSeq* predictions are performed (each position treated as a 5-mer) and is varied from 1 base up to 21 base window, as specified in a given situation.

Supplementary method SM2:

Training models: SVR, MLR and Elastic Nets:

Three different machine-learning models are used in this work selected subject to their suitability in different contexts, as follows.

1. A support vector regression (SVR) model with radial basis function (RBF) kernel was used to develop the *DynaSeq* core predictor, with DNA sequences from the MD trajectory data as inputs and the corresponding ensemble bin populations as an output. SVR with a radial basis function allows for non-additive contributions of various features hence this model was selected to account for sequence neighbors impacting the conformational ensembles in a non-additive manner.
2. For the predictions of DREAM5 sequence specificity values over training and test data, a Multiple Linear Regression (MLR) model was used considering the fact that the entire ensemble for all base positions within the input sequences is quite large and we do not wish to increase its complexity by introducing non-additive terms. This model was not cross-validated during training as the test data provides for the blind benchmark and was not utilized during the training of models.
3. For predicting TF binding sites compared to control, we opted for a regularized logistic regression. **Lasso and ridge regression** are well-known techniques to avoid overfitting in generalized linear models. **Elastic net models** provide a balance between the two regularization models (28) and have been successfully utilized in addressing important biological problems (e.g. (29)). Since, the objective of TF binding site classification is to avoid overfitting and estimating performance levels to allow comparison of various models, an elastic net regularized model with alpha=0.1 was utilized for classifying TF binding sites (30). During training, *glmnet* attempts to find the optimum model by trying different values of lambda and such models produce different prediction performances. We have selected a model with highest AUC score in each case.

Supplementary method SM3:

Benchmarking *DynaSeq* with known structures in PDB

All high resolution (≤2.5Å) free DNA structures (without protein or RNA) available in the PDB were compiled and those with structural or sequence anomalies (e.g. quadruplex, triplex and modified base) were removed, leaving 115 entries (32). For each of these entries from the PDB, additional 1000 random sequences of the same length were generated and conformational ensembles were predicted for each one of them. The ability of *DynaSeq* to predict the sequence specificity of a given structure is determined by evaluating the agreement between observed conformational parameters on the one hand and those predicted for native and random genomic sequence on the other (native sequence refers to the one observed in the PDB file for the given structure under

consideration). If the agreement on predicted conformations is better for the native sequence compared with random sequences, favorable sequence-specificity is established. The agreement between a single pair of structures is computed by first taking the Pearson's correlation coefficient between predicted and observed parameters one at a time. An average of 12 correlation coefficients obtained in this way is taken as the final measure of agreement between two structures. In this way an average correlation coefficients for each comparison (one native and 100 random) are obtained. The superiority of native predicted/observed agreement over random sequences is measured by taking the Z-score of these averaged Pearson's correlations.

$$Z = \frac{<R_{op}^{r}> - R_{op}^{o}}{\sigma(R_{op}^{r})} \qquad (1)$$

where R_{op}^{o} is the average Pearson correlation difference between observed *(o)* and predicted *(p)* structures of DNA sequence when the sequence being used for prediction is the same as observed in the PDB data and R_{op}^{r} are the set of averaged correlation coefficients for a random sequences (*r)*, which have the same length as (*o)*. Denominator represents the standard deviation and <X> measures the average of values contained in X. The Z-score is converted to a p-value using a normal distribution function.

$$p = pnorm(Z) \qquad (2)$$

To reflect the true nature of *DynaSeq* predictions, comparisons were made between the averages of predicted *individual* conformational parameters with those observed in the crystal structure. In addition, a complete atomic structure was modeled by using the conformational parameter values corresponding to the conformational bins, *most populated* in the predicted ensemble*.* Using the most populated bins instead of averages from each bin makes it more likely that the final three-dimensional structure represents a real conformation allowing structural alignment in a traditional manner and computing RMSD, which may be easier to understand. Ensemble populations for all these sequences were time-averaged and 12 conformational parameters are predicted at each position and the agreements between predicted and observed structures for native and random sequences are computed by Pearson's correlation for each parameter independently and averaging all these values. Z-score is used to quantify the degree of increase in the Pearson's correlation for the native sequence compared to the random set. By definition a higher negative Z-score indicates a favorable prediction for the native sequence compared to a random one.

Supplementary method SM4:

Treatment of DREAM5 data sets:

DREAM5 data consist of pairs of experiments reporting binding activities of 86 mouse TFs for 32896 *De Bruijn* DNA sequences (8-mers each) (33). Each pair

consists of a training set and a test set on binding affinities measured from protein binding arrays. We developed multiple linear regression (MLR) models over these data in two ways. First, the TFs were annotated to be binding or non-binding to each DNA sequence at a threshold of Z-score=4 (which is consistent with the class labels used in the original competition). MLR models were then trained to predict the binding class label from the *DynaSeq*-predicted and *DNAshape*-predicted features for the entire sequence. Leaving 2-bases on either terminal, the predictions are made for the remaining six positions and all predicted values are concatenated to form the inputs for the MLR model. Models are trained over TF data labeled as "*HK design*" and tested on one labeled as "*ME design*" in each case. MLR returns numerical values, which using various cutoffs were utilized to plot ROC curves and evaluate AUC under each feature set choice. Input feature sets used in this work consist of (1) four *DNAshape* features (2) *DynaSeq*-predicted values of the same four features that appear in *DNAshape* (named as *DynaSeq.4D* in this paper) and (3) *DynaSeq.60D* which uses the entire set of 60 features predicted by *DynaSeq*. Note that these numbers (4 or 60) refer to a single base position in one of the De Bruijn Sequences and the actual number of features input to MLR model is 6 times this number.

In addition to AUC values, MLRs were also separately trained to predict raw scores available from DREAM5 data. This allows us to give a more quantitative estimate of affinity prediction performance, which is measured by a Pearson's correlation between predicted and observed values (in the test data).

Supplementary Results:

Supplementary Result SR1:

Benchmarking with DREAM5 TF-binding specificity data:

As a final benchmark to compare *DNAshape* and *DynaSeq* predictions, we used the data sets from DREAM5 competition. This data consists of 87 TFs with paired set of independently determined binding sites for each of them. Models are trained on one set and tested on the other within a pair using MLR. We computed the AUC and Pearson correlation between predictions by *DNAshape* features. For *DynaSeq*, we trained our models in two ways. First, we used predictions of the same four features as used in *DNAshape* for training the classifier (*DynaSeq.4D*) and compared if the two feature sets differ. Our prediction results for revealed that both *DNAshape* and *DynaSeq* performed similarly (Pearson correlation between performances in all 87 TFs is close to 0.9) when similar features are used for training these models (Supplementary Figure SF2(a)). Even though the prediction results from *DNAshape* features and *DynaSeq.4D* features are similar, the AUC of the consensus shows significant improvement over *DNAshape*-based results with a mean AUC rising from 75.68% to 77.60% (p-value from t-test=0.0015, Supplementary Figure SF2(b). When, all *DynaSeq* features are used

to train and evaluate DREAM5 binding site data, the binding site data with DynaSeq.60D (all *DynaSeq* features) could be modeled with an AUC as high as 93.9% (Supplementary Figure SF2 (c-d)). AUC values of more than 90% still do not translate into a very high degree of Pearson's correlation which remain limited to the average values of 0.46, 0.43 and 0.60 for DNAshape, DynaSeq.4D and DynaSeq.60D based models (Supplementary Table ST3).

Supplementary Figures:

Supplementary Figure SF1. Basic statistics of *DynaSeq* ensemble bins for 12 conformational parameters. (a) Range of observed values for each conformational parameter and coverage of values within each of the five ensemble bins. These represent average occupancy of a ensemble bin for all base positions in all tetramers. Within each position of a tetramer, populations differ from one another and the same is shown in (b) as standard deviations for all such measurements. Ensemble bin populations by grouping nucleotide positions by their base name and triplet are shown in (c) and (d) respectively.

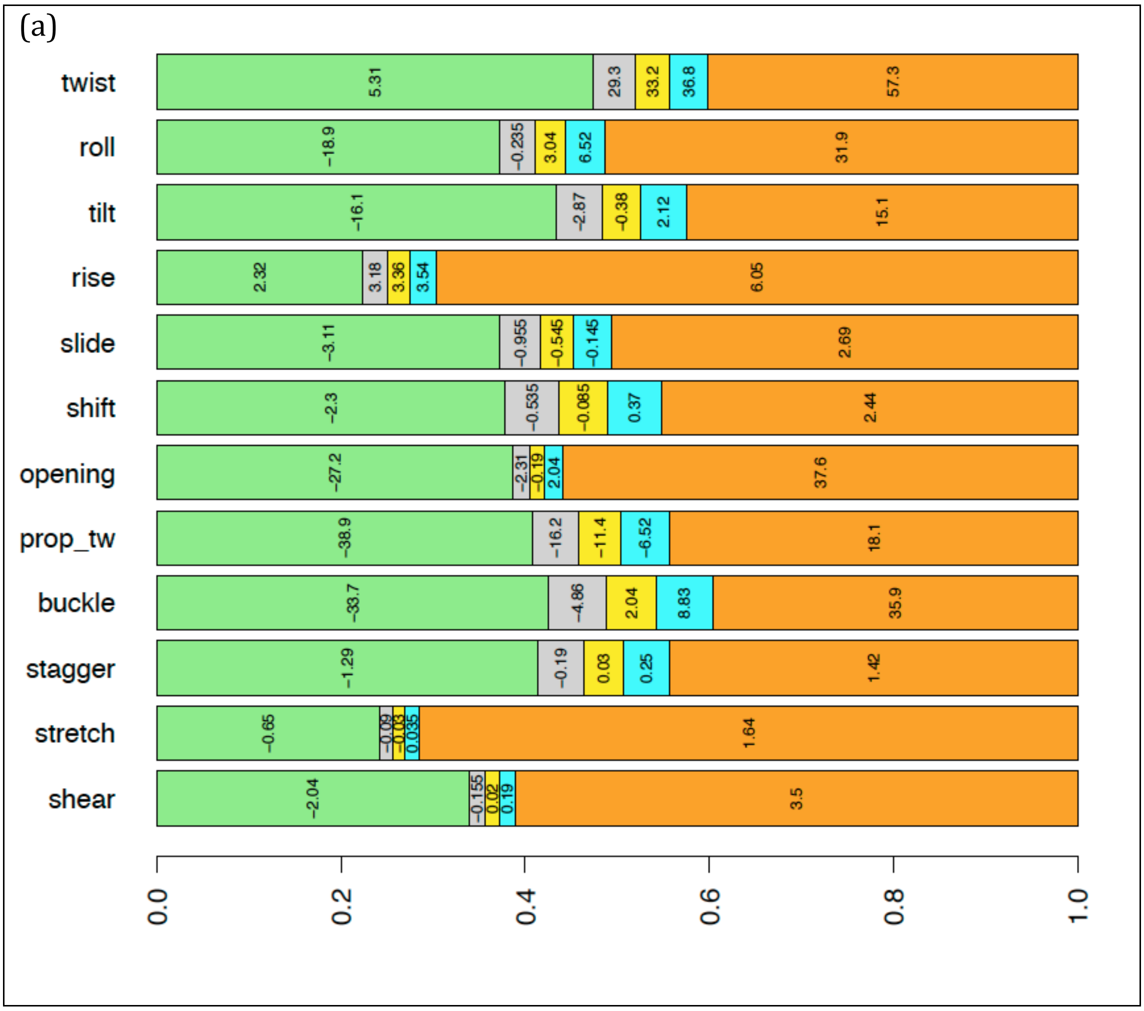

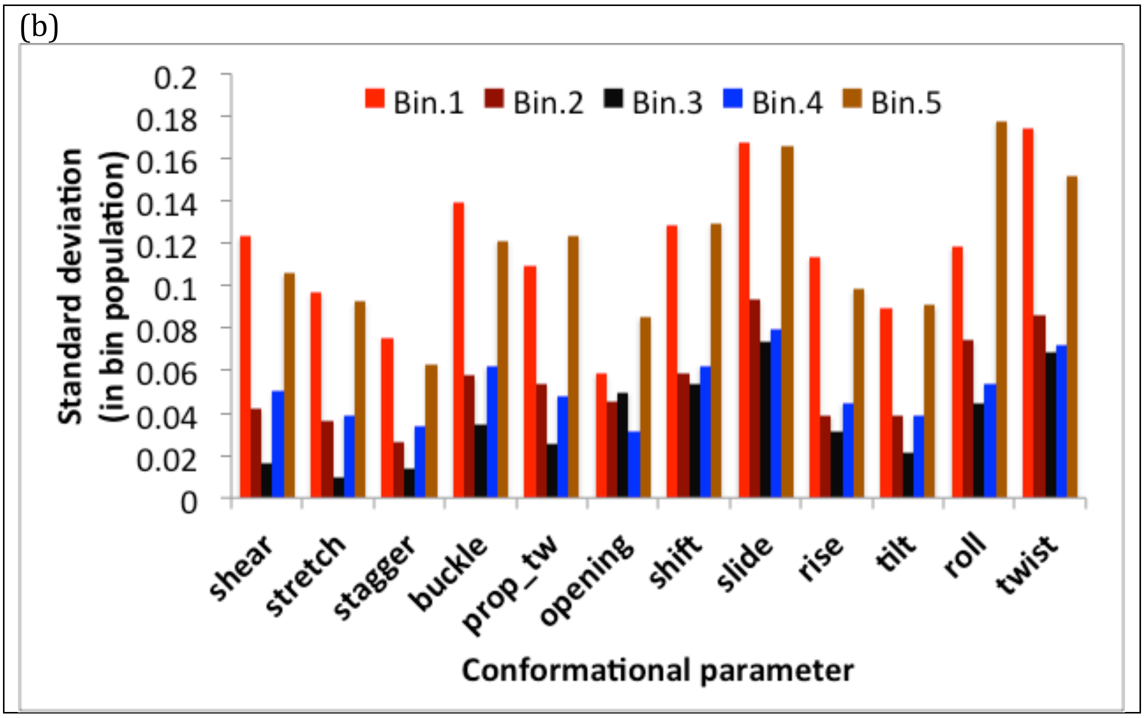
(b)
Bin.1
Bin.2
Bin.3
Bin.4
Bin.5
Standard deviation (in bin population)
0.2
0.18
0.16
0.14
0.12
0.1
0.08
0.06
0.04
0.02
0
shear
stretch
stagger
buckle
prop_tw
opening
shift
slide
rise
tilt
roll
twist
Conformational parameter

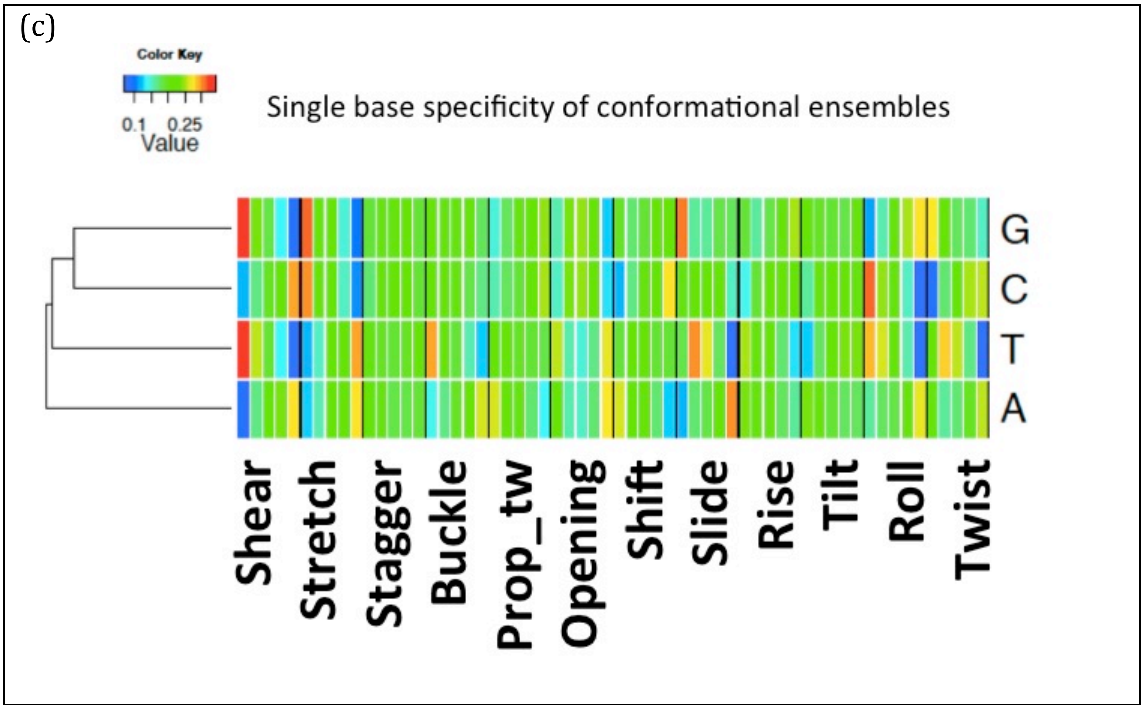
(c)
Color Key
0.1
0.25
Value
Single base specificity of conformational ensembles
G
C
T
A
Shear
Stretch
Stagger
Buckle
Prop_tw
Opening
Shift
Slide
Rise
Tilt
Roll
Twist

(d)

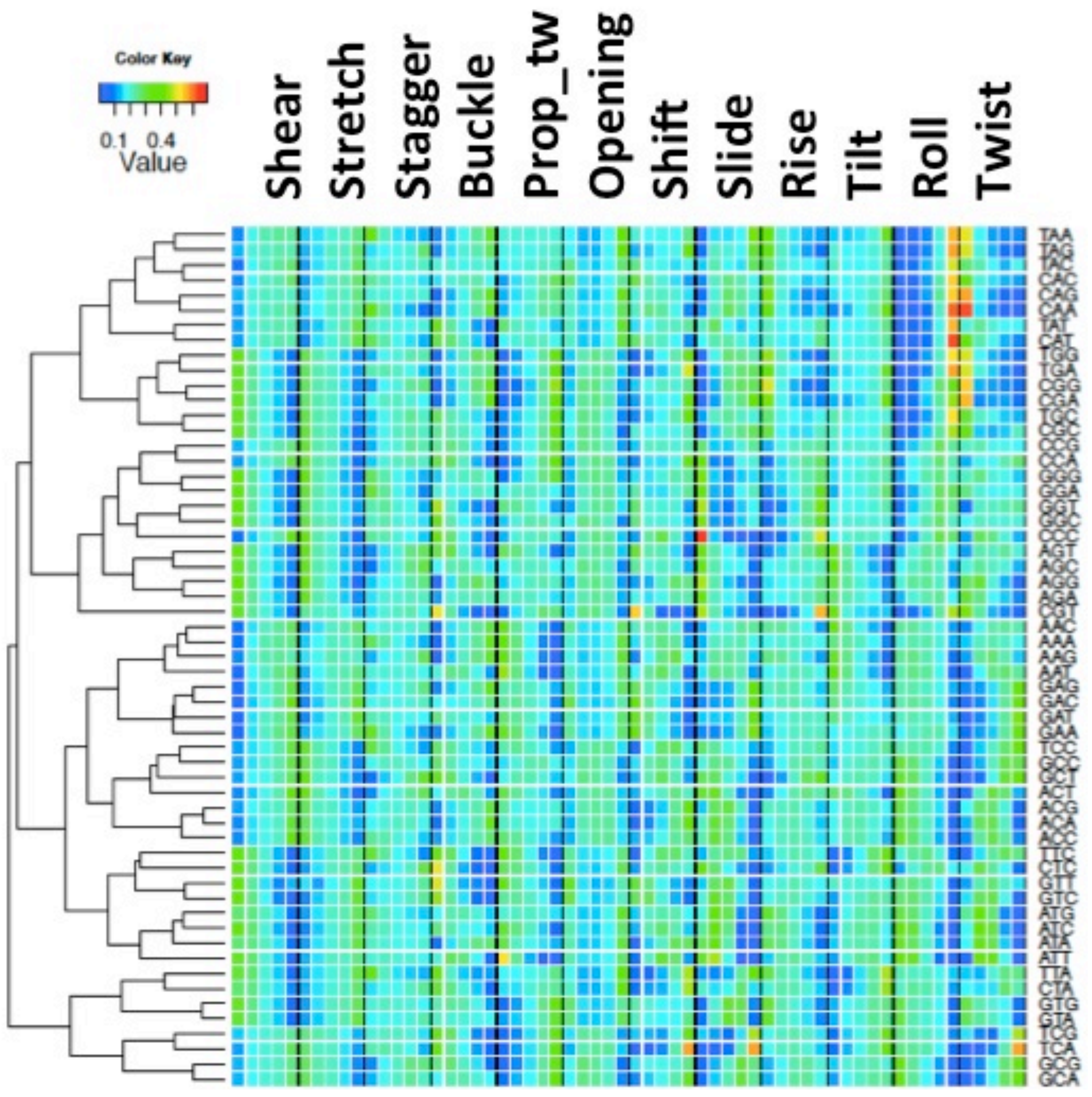

Color Key
0.1 0.4
Value
Shear
Stretch
Stagger
Buckle
Prop_tw
Opening
Shift
Slide
Rise
Tilt
Roll
Twist
TAA
TAG
TAC
CAC
CAG
CAA
TAT
CAT
TGG
TGA
CGG
CGA
TGC
CGC
CCG
CCA
GGG
GGA
GGT
GGC
CCC
AGT
AGC
AGG
AGA
CGT
AAC
AAA
AAG
AAT
GAG
GAC
GAT
GAA
TCC
GCC
GCT
ACT
ACG
ACA
ACC
TTC
CTC
GTT
GTC
ATG
ATC
ATA
ATT
TTA
CTA
GTG
GTA
TCG
TCA
GCG
GCA

Supplementary Figure SF2. Comparison between *DNAshape* and *DynaSeq* performance on DREAM5 specificty prediction (detailed results for each of the transcription factor are provided in supplementary table ST3). *DynaSeq.4D* refers to the four *DNAshape* features predicted by *DynaSeq*, whereas *DynaSeq* features include all the 65 features. (a) Comparison between AUC results obtained by *DNAshape* and *DynaSeq* using similar features predicted by either of them (b) When results are predicted by *DNAshape* and *DynaSeq* (similar features, as in (a)) and consensus is taken over the two models, the AUC of the consensus shows significant imporvement over *DNAshape*-based results with a mean AUC rising from 75.68% to 77.60% (p-value from t-test=0.0015). The scatterplot shows that the gain is more obvious for TFs poorly modeled by *DNAshape*. (c) and (d) compare the predictions from full set of *DynaSeq* features with the best of prediction scores obtained from *DNAshape* or corresponding 4D features from *DynaSeq*. These plots clearly establish that *DynaSeq* feature set outperformed *DNAshape* features by raising the average AUC to 93% (a large gain of 18% AUC).

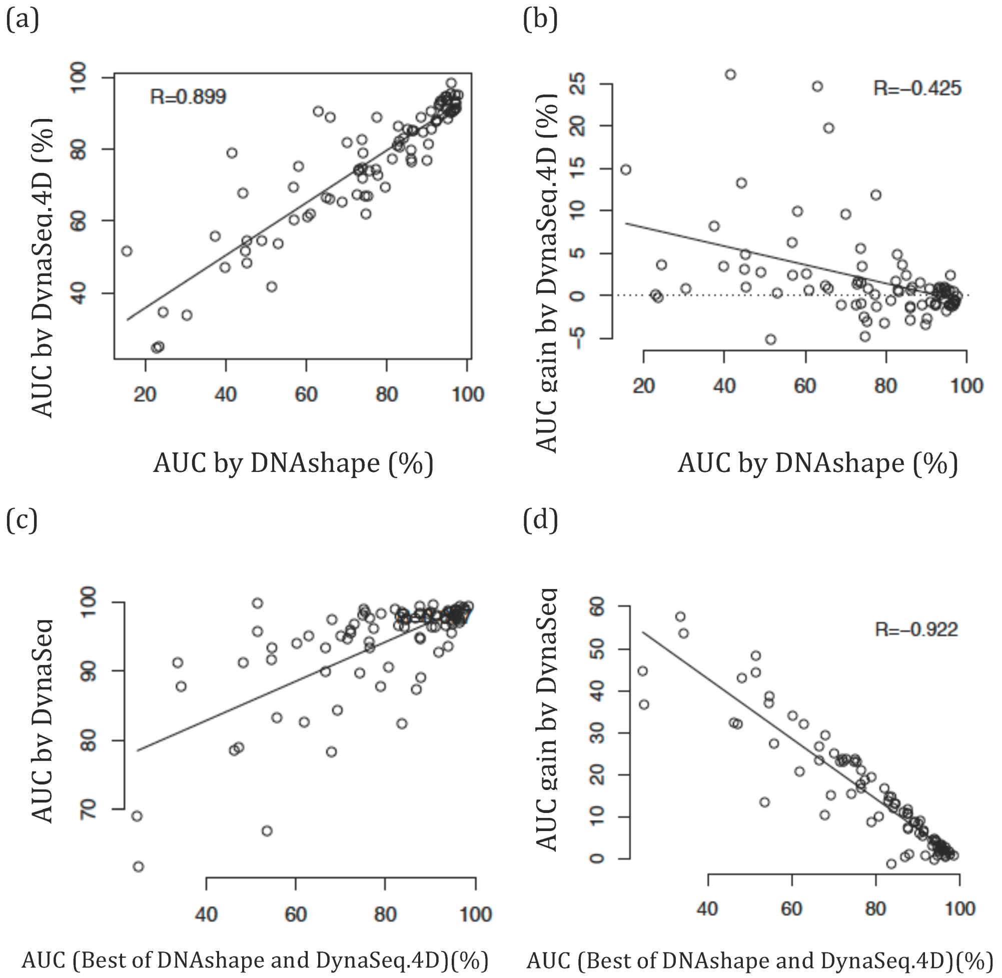

Supplementary Tables

Supplementary Table ST1: Base pair and base-step parameters, representing DNA structure, considered in this work (Minor groove width not shown).

Parameter type		Name	Description	Figure
Base pair	Translational	Shear	Complementary bases' lateral displacement normal to helical axis	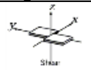
		Stretch	Complementary bases' mutual displacement normal to helical axis	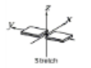
		Stagger	Complementary bases' mutual displacement normal to helical axis	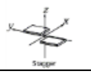
	Rotational	Buckle	Displacement bending complementary base-plane	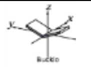
		Propeller	Displacement twisting complementary base-plane	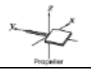
		Opening	Rotational displacement of bases within the base-plane	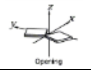
Base-step	Translational	Shift	Displacement of one base pair relative to the other normal to helical axis and axis joining complementary bases	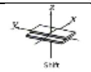
		Slide	Displacement of one base pair relative to the other along axis joining complementary bases	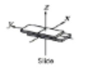
		Rise	Displacement of one base pair relative to the other along helical axis	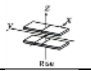
	Rotational	Tilt	Change in angle between complementary base-pair axis of adjacent pairs normal to helical axis	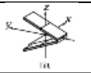
		Roll	Change in angle normal to the helical axis and complementary base-pair axis of adjacent pairs	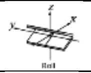
		Twist	Change in angle between complementary base-pair axis of adjacent pairs around the helical axis	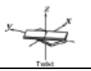

Supplementary Table ST2: Conformational specificty of 115 DNA sequences avialble from Protein Data Bank (PDB). *DynaSeq* conformational parameters are predicted for DNA sequence in the PDB and then another 1000 randomly generated sequences of the same length. RMSD with the structures observed in PDB is computed for all of them and Z-score for the real sequence versus the random set is computed. P-values are obtained by using Pnorm of the Z-score.

**PDB ID**	Z-score	P-value	**PDB ID**	Z-score	P-value	**PDB ID**	Z-score	P-value
**102d**	-3.65	1.3E-04	**1m6f**	-3.31	4.6E-04	**3co3**	-1.98	2.4E-02
**113d**	-3.33	4.3E-04	**1prp**	-3.13	8.8E-04	**3oie**	-2.66	3.9E-03
**109d**	-3.12	9.1E-04	**1puy**	-1.60	5.5E-02	**3qk4**	-1.15	1.3E-01
**119d**	-1.76	3.9E-02	**1qv4**	-2.56	5.2E-03	**3u05**	-3.17	7.6E-04
**121d**	-1.37	8.5E-02	**1qv8**	-2.92	1.7E-03	**3u08**	-3.74	9.0E-05
**127d**	-2.24	1.2E-02	**1s2r**	-3.68	1.2E-04	**3u0u**	-3.43	3.0E-04
**129d**	-2.60	4.7E-03	**1vtj**	-1.69	4.5E-02	**3u2n**	-3.54	2.0E-04
**166d**	-2.94	1.7E-03	**1vzk**	-4.38	5.8E-06	**3v9d**	-2.75	3.0E-03
**194d**	-2.97	1.5E-03	**1zje**	-1.95	2.5E-02	**423d**	-3.43	3.0E-04
**195d**	-3.11	9.4E-04	**1zjf**	-2.20	1.4E-02	**425d**	-0.67	2.5E-01
**1bna**	-3.72	9.8E-05	**1zjg**	-2.47	6.7E-03	**428d**	-3.15	8.2E-04
**1d28**	-1.67	4.7E-02	**1zph**	-3.87	5.3E-05	**442d**	-3.44	3.0E-04
**1d29**	-2.60	4.6E-03	**1zpi**	-3.97	3.7E-05	**443d**	-3.36	3.9E-04
**1d30**	-2.83	2.3E-03	**227d**	-3.20	6.8E-04	**444d**	-2.93	1.7E-03
**1d43**	-3.65	1.3E-04	**263d**	-2.65	4.0E-03	**445d**	-3.09	1.0E-03
**1d44**	-3.33	4.3E-04	**264d**	-3.68	1.2E-04	**447d**	-2.79	2.7E-03
**1d45**	-2.98	1.4E-03	**287d**	-3.44	2.9E-04	**448d**	-3.03	1.2E-03
**1d46**	-2.76	2.9E-03	**289d**	-2.28	1.1E-02	**449d**	-3.26	5.6E-04
**1d63**	-3.11	9.4E-04	**296d**	-2.20	1.4E-02	**453d**	-3.82	6.6E-05
**1d64**	-2.87	2.1E-03	**298d**	-2.29	1.1E-02	**455d**	-4.10	2.1E-05
**1d65**	-3.09	1.0E-03	**2b0k**	-3.29	5.1E-04	**4agz**	-4.03	2.7E-05
**1d86**	-3.30	4.9E-04	**2b3e**	-3.51	2.2E-04	**4ah0**	-3.94	4.1E-05
**1d98**	-1.80	3.6E-02	**2bna**	-3.79	7.7E-05	**4ah1**	-3.10	9.8E-04
**1d99**	-2.42	7.8E-03	**2d47**	0.64	7.4E-01	**4c64**	-3.92	4.3E-05
**1dc0**	-1.73	4.2E-02	**2dbe**	-3.60	1.6E-04	**4l24**	-2.62	4.3E-03
**1dn9**	-2.52	5.9E-03	**2dnd**	-1.86	3.2E-02	**4okl**	-2.71	3.4E-03
**1dne**	-1.75	4.0E-02	**2dyw**	-3.51	2.3E-04	**4u8a**	-3.96	3.8E-05
**1dnh**	-2.34	9.6E-03	**2gvr**	-3.69	1.1E-04	**4u8b**	-3.63	1.4E-04
**1eel**	-2.14	1.6E-02	**2gyx**	-2.97	1.5E-03	**4u8c**	-3.62	1.5E-04
**1fmq**	-3.41	3.3E-04	**2i2i**	-3.59	1.6E-04	**4z4b**	-4.18	1.5E-05
**1fms**	-3.55	1.9E-04	**2i5a**	-2.97	1.5E-03	**5bna**	-3.35	4.0E-04
**1fq2**	-3.49	2.4E-04	**2nlm**	-2.74	3.1E-03	**5dam**	-3.50	2.4E-04
**1ftd**	-3.59	1.7E-04	**302d**	-3.38	3.6E-04	**5et9**	-0.89	1.9E-01
**1hq7**	-2.49	6.3E-03	**303d**	-3.58	1.7E-04	**5ezf**	-3.07	1.1E-03
**1ihh**	-3.03	1.2E-03	**311d**	-3.24	6.0E-04	**7bna**	-3.20	6.8E-04
**1jgr**	-4.06	2.4E-05	**328d**	-2.68	3.7E-03	**8bna**	-2.82	2.4E-03
**1lex**	-3.18	7.3E-04	**355d**	-3.48	2.5E-04	**9bna**	-3.84	6.1E-05
**1ley**	-3.19	7.0E-04	**360d**	-3.25	5.8E-04			
**1lp7**	-3.08	1.0E-03	**3bse**	-1.21	1.1E-01			

Supplementary Table ST3: DREAM5 specificty prediction results

TFID	Trained	Tested	rr.ds	rr.d4	rr.d60	rr.cons.d4.ds	auc.ds	auc.d4	auc.d60	auc.cons.d4.ds
1	Srebf1_HK.design	Srebf1_ME.design	0.54	0.52	0.60	0.09	86.0	79.7	97.8	84.7
2	Klf12_HK.design	Klf12_ME.design	0.42	0.34	0.42	0.02	44.2	68.0	97.5	57.5
3	Sp1_HK.design	Sp1_ME.design	0.62	0.59	0.66	0.04	61.0	61.9	82.7	61.6
4	Dnajc21_HK.design	Dnajc21_ME.design	0.27	0.28	0.52	-0.07	24.4	34.4	87.9	28.1
5	Atf4_HK.design	Atf4_ME.design	0.65	0.62	0.68	0.05	86.2	85.4	87.4	86.9
6	Gata4_HK.design	Gata4_ME.design	0.59	0.60	0.72	0.17	86.6	85.3	98.4	87.7
7	Nr2c1_HK.design	Nr2c1_ME.design	0.37	0.37	0.54	0.00	45.2	54.7	93.5	50.0
8	Foxp2_HK.design	Foxp2_ME.design	0.77	0.75	0.78	0.11	97.1	93.1	97.5	96.6
9	Zscan10_HK.design	Zscan10_ME.design	0.40	0.32	0.46	-0.01	51.4	41.6	78.6	46.3
10	Sox10_HK.design	Sox10_ME.design	0.73	0.67	0.79	0.20	94.6	91.5	98.6	94.2
11	Ar_HK.design	Ar_ME.design	0.55	0.51	0.62	0.08	72.9	73.9	89.8	74.2
12	Nr2f6_HK.design	Nr2f6_ME.design	0.10	0.12	0.40	0.10	73.9	72.0	96.8	73.0
13	Zkscan1_HK.design	Zkscan1_ME.design	0.72	0.61	0.80	0.11	89.9	76.8	97.6	86.6
14	Zkscan5_HK.design	Zkscan5_ME.design	0.70	0.68	0.71	0.11	93.0	92.7	93.7	93.9
15	Esrrb_HK.design	Esrrb_ME.design	0.66	0.64	0.68	0.02	56.8	69.3	84.3	63.1
16	Snai1_HK.design	Snai1_ME.design	0.35	0.35	0.63	0.15	82.8	86.5	99.4	87.6
17	Klf9_HK.design	Klf9_ME.design	0.66	0.64	0.48	0.05	73.9	74.9	98.5	75.5
18	Ahctf1_HK.design	Ahctf1_ME.design	0.72	0.66	0.79	0.24	97.7	95.4	98.9	97.7
19	Zbtb1_HK.design	Zbtb1_ME.design	0.49	0.43	0.56	0.08	79.6	69.5	94.3	76.4
20	Zscan10_HK.design.1	Zscan10_ME.design.1	0.48	0.45	0.56	0.00	45.3	48.2	91.2	46.4
21	Zscan10_HK.design.2	Zscan10_ME.design.2	0.24	0.22	0.41	-0.09	23.5	24.9	61.7	23.3
22	Zfp300_HK.design	Zfp300_ME.design	0.69	0.65	0.71	0.07	83.0	82.3	82.4	83.7
23	Sox14_HK.design	Sox14_ME.design	0.72	0.68	0.76	0.14	92.4	88.1	98.0	91.4
24	Zfp263_HK.design	Zfp263_ME.design	0.30	0.35	0.50	0.03	60.3	61.2	95.1	62.9
25	Atf3_HK.design	Atf3_ME.design	0.55	0.52	0.63	0.09	73.8	82.9	96.6	79.3
26	Zic5_HK.design	Zic5_ME.design	0.14	0.20	0.40	-0.02	37.4	55.8	83.3	45.6
27	Egr3_HK.design	Egr3_ME.design	0.16	0.28	0.58	0.17	65.9	89.0	97.7	85.6
28	Irf2_HK.design	Irf2_ME.design	0.49	0.44	0.59	0.18	90.3	81.4	95.0	87.6
29	Nkx2.9_HK.design	Nkx2.9_ME.design	0.52	0.45	0.69	0.09	74.8	62.1	95.1	70.1
30	Foxg1_HK.design	Foxg1_ME.design	0.59	0.52	0.64	0.17	96.9	91.5	98.0	95.9
31	Xbp1_HK.design	Xbp1_ME.design	0.63	0.59	0.73	0.16	88.5	88.9	98.6	90.1
32	Znf740_HK.design	Znf740_ME.design	0.21	0.25	0.44	-0.03	15.5	51.5	99.9	30.3
33	Mlx_HK.design	Mlx_ME.design	0.27	0.23	0.43	0.10	97.1	92.9	99.5	96.6
34	Tfec_HK.design	Tfec_ME.design	0.45	0.40	0.51	0.11	86.0	77.4	97.5	84.5

35	Zfx_HK.design	Zfx_ME.design	0.07	0.14	0.69	0.18	63.0	90.6	99.7	87.6
36	Nr2e1_HK.design	Nr2e1_ME.design	0.31	0.35	0.69	0.13	70.1	82.1	98.9	79.7
37	Nhlh2_HK.design	Nhlh2_ME.design	0.57	0.52	0.62	0.03	68.9	65.3	78.3	67.9
38	Nfil3_HK.design	Nfil3_ME.design	0.66	0.62	0.73	0.19	93.0	92.3	97.8	93.4
39	Foxj2_HK.design	Foxj2_ME.design	0.73	0.67	0.74	0.17	97.2	91.7	98.7	96.4
40	Sdccag8_HK.design	Sdccag8_ME.design	0.66	0.58	0.82	0.28	96.9	95.3	99.3	97.4
41	Mecp2_HK.design	Mecp2_ME.design	0.72	0.63	0.81	0.16	91.1	85.8	96.3	90.3
42	Junb_HK.design	Junb_ME.design	0.70	0.67	0.73	0.09	90.9	90.8	92.8	91.8
43	Zfp202_HK.design	Zfp202_ME.design	0.12	0.30	0.59	0.27	96.0	98.5	99.4	98.5
44	Gmeb2_HK.design	Gmeb2_ME.design	0.44	0.42	0.76	0.32	93.7	89.7	98.8	93.9
45	Klf8_HK.design	Klf8_ME.design	0.11	0.22	0.45	0.17	77.5	89.1	98.3	89.4
46	Sox3_HK.design	Sox3_ME.design	0.69	0.64	0.77	0.16	92.3	88.0	98.1	91.4
47	Egr2_HK.design	Egr2_ME.design	0.19	0.24	0.53	0.06	41.5	79.0	98.3	67.5
48	Zfp637_HK.design	Zfp637_ME.design	0.65	0.61	0.72	0.08	93.8	93.6	95.6	94.7
49	Dmrtc2_HK.design	Dmrtc2_ME.design	0.78	0.75	0.80	0.16	95.8	95.5	97.1	96.5
50	Cebpb_HK.design	Cebpb_ME.design	0.53	0.46	0.64	0.10	86.1	76.4	97.9	83.2
51	Zfp3_HK.design	Zfp3_ME.design	0.46	0.40	0.58	0.09	81.3	77.2	90.7	80.7
52	Prdm11_HK.design	Prdm11_ME.design	0.32	0.29	0.63	0.19	85.1	85.7	98.4	87.5
53	P42pop_HK.design	P42pop_ME.design	0.32	0.31	0.57	0.09	73.1	74.3	98.1	74.9
54	Rora_HK.design	Rora_ME.design	0.36	0.34	0.50	0.00	44.9	51.5	95.8	48.1
55	Nr4a2_HK.design	Nr4a2_ME.design	0.55	0.54	0.65	0.06	65.0	66.6	93.3	66.2
56	Nr5a2_HK.design	Nr5a2_ME.design	0.33	0.32	0.42	-0.02	39.8	47.2	79.1	43.3
57	Rorb_HK.design	Rorb_ME.design	0.05	0.09	0.49	0.04	56.9	60.2	94.1	59.4
58	Foxo6_HK.design	Foxo6_ME.design	0.62	0.55	0.68	0.19	95.7	90.5	97.4	94.6
59	Esr1_HK.design	Esr1_ME.design	0.09	0.11	0.50	0.01	49.0	54.6	91.6	51.8
60	Rfx7_HK.design	Rfx7_ME.design	0.43	0.38	0.60	0.07	72.6	67.6	94.7	71.5
61	Sp140_HK.design	Sp140_ME.design	0.60	0.59	0.68	0.08	74.1	78.9	87.7	77.6
62	Mybl2_HK.design	Mybl2_ME.design	0.29	0.26	0.53	0.13	83.2	80.6	98.5	83.7
63	Foxo4_HK.design	Foxo4_ME.design	0.37	0.31	0.50	0.17	96.7	91.1	98.1	95.8
64	Foxo3_HK.design	Foxo3_ME.design	0.43	0.36	0.48	0.15	96.4	90.9	98.1	95.2
65	Tbx2_HK.design	Tbx2_ME.design	0.40	0.32	0.55	0.08	74.6	66.9	95.9	72.1
66	Tbx5_HK.design	Tbx5_ME.design	0.52	0.35	0.36	0.11	84.1	83.1	94.7	87.7
67	Tbx20_HK.design	Tbx20_ME.design	0.28	0.23	0.36	0.05	77.7	72.8	93.3	76.5
68	Foxc2_HK.design	Foxc2_ME.design	0.69	0.63	0.73	0.20	96.9	93.6	97.8	96.4
69	Nr2f1_HK.design	Nr2f1_ME.design	0.25	0.28	0.64	0.19	77.3	74.4	96.1	77.4
70	Pou1f1_HK.design	Pou1f1_ME.design	0.66	0.64	0.77	0.22	94.5	94.9	98.7	95.5

71	Pou3f1_HK.design	Pou3f1_ME.design	0.67	0.64	0.76	0.22	94.8	94.7	99.0	95.6
72	Tbx1_HK.design	Tbx1_ME.design	0.64	0.59	0.64	0.10	89.0	84.7	89.0	87.9
73	Dbp_HK.design	Dbp_ME.design	0.65	0.61	0.64	0.12	94.8	93.8	97.2	95.1
74	Esrrg_HK.design	Esrrg_ME.design	0.14	0.15	0.41	0.08	65.8	66.2	90.0	66.6
75	Foxo1_HK.design	Foxo1_ME.design	0.57	0.52	0.63	0.19	95.9	90.3	96.8	94.9
76	Foxp1_HK.design	Foxp1_ME.design	0.59	0.53	0.66	0.17	95.1	88.5	96.7	93.4
77	Sox6_HK.design	Sox6_ME.design	0.60	0.54	0.65	0.14	92.2	87.6	96.5	91.1
78	Tcf3_HK.design	Tcf3_ME.design	0.08	0.11	0.34	0.04	58.1	75.1	99.0	68.1
79	Tbx4_HK.design	Tbx4_ME.design	0.58	0.48	0.48	0.09	82.5	81.0	96.3	84.2
80	Mzf1_HK.design	Mzf1_ME.design	-0.08	-0.08	0.18	-0.05	30.4	33.6	91.3	31.3
81	Mzf1_HK.design.1	Mzf1_ME.design.1	0.12	0.11	0.28	-0.09	22.9	24.6	69.1	23.0
82	Zscan20_HK.design	Zscan20_ME.design	0.20	0.18	0.26	0.01	53.0	53.6	66.9	53.4
83	Tbx3_HK.design	Tbx3_ME.design	0.61	0.55	0.68	0.07	75.3	66.8	95.5	72.3
84	Rarg_HK.design	Rarg_ME.design	0.15	0.18	0.49	0.11	75.6	74.2	97.7	76.5
85	Oct1_HK.design	Oct1_ME.design	0.52	0.50	0.69	0.21	93.3	93.6	98.6	94.3
86	Pit1_HK.design	Pit1_ME.design	0.56	0.53	0.71	0.23	95.0	94.2	98.7	95.5
